# Protonated C_3_N_4_ Nanosheets for Enhanced Energy Storage in Symmetric Supercapacitors through Hydrochloric Acid Treatment

**DOI:** 10.1021/acsomega.3c06747

**Published:** 2024-02-28

**Authors:** Mahalakshmi Subbiah, Annalakshmi Mariappan, Anandhakumar Sundaramurthy, Sabarinathan Venkatachalam, Rajasekaran Thanjavur Renganathan, Nishakavya Saravanan, Sudhagar Pitchaimuthu, Nagarajan Srinivasan

**Affiliations:** † Department of Renewable Energy Science, Manonmaniam Sundaranar University, Tirunelveli 627012, India; ‡ Laboratory of Electrochemical Interfaces, Department of Chemistry, Manonmaniam Sundaranar University, Tirunelveli 627012, India; § Biomaterials Research Laboratory, Department of Chemical Engineering, SRM Institute of Science and Technology, Kattankulathur 603203, Tamil Nadu India; ∥ Department of Physics, Manonmaniam Sundaranar University, Tirunelveli 627012, India; ⊥ Department of Physics and Nanotechnology, SRM Institute of Science and Technology, Kattankulathur603203, Tamil Nadu, India; # Research Centre for Carbon Solutions (RCCS), Institute of Mechanical, Processing and Energy Engineering, School of Engineering and Physical Sciences, Heriot-Watt University, Edinburgh EH14 4AS, U.K.

## Abstract

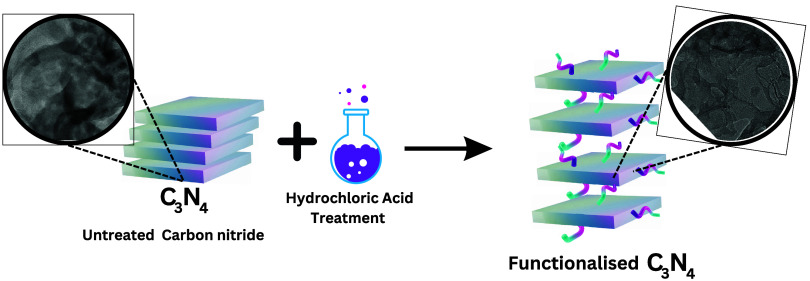

Next-generation electrochemical energy storage materials are essential in delivering high power for long periods of time. Double-layer carbonaceous materials provide high power density with low energy density due to surface-controlled adsorption. This limitation can be overcome by developing a low-cost, more abundant material that delivers high energy and power density. Herein, we develop layered C_3_N_4_ as a sustainable charge storage material for supercapacitor applications. It was thermally polymerized using urea and then protonated with various acids to enhance its charge storage contribution by activating more reaction sites through the exfoliation of the C–N framework. The increased electron-rich nitrogen moieties in the C–N framework material lead to better electrolytic ion impregnation into the electrode, resulting in a 7-fold increase in charge storage compared to the pristine material and other acids. It was found that C_3_N_4_ treated with hydrochloric acid showed a very high capacitance of 761 F g^–1^ at a current density of 20 A g^–1^ and maintained 100% cyclic retention over 10,000 cycles in a three-electrode configuration, outperforming both the pristine material and other acids. A symmetric device was fabricated using a KOH/LiI gel-based electrolyte, exhibiting a maximum specific capacitance of 175 F g^–1^ at a current density of 1 A g^–1^. Additionally, the device showed remarkable power and energy density, reaching 600 W kg^–1^ and 35 Wh kg^–1^, with an exceptional cyclic stability of 60% even after 5000 cycles. This study provides an archetype to understand the underlying mechanism of acid protonation and paves the way to a metal–carbon-free environment.

## 1 Introduction

Because of the current energy crisis and the need for sustainable energy, supercapacitors have gained considerable attention as energy storage devices due to their high power and energy densities and excellent cyclic stability.^1−3^ Currently, researchers are focusing on 3D porous carbon and other sheet-like carbon materials for advanced energy storage systems.^4−6^ Graphene is a two-dimensional layered material with good electrical and ionic conductivity and superior chemical stability.^7^ However, it has a low productivity, which could limit its application.^8^ Graphitic carbon nitride (g-C_3_N_4_) is a stable allotrope that is obtained through the thermal polymerization of nitrogen precursors such as melamine, urea, thiourea, and ammonium thiocyanate.^9^ This material is widely used as a catalyst and reactant in photo-electrochemical water splitting and environmental remediation applications.^10−14^ It has a two-dimensional layered structure with weak van der Waals forces between the layers, where tri-s-triazine is bounded by tertiary amines.^15−17^ Its conjugated structure with sp_2_-hybridized bonds between carbon and nitrogen in each layer gives it unique properties and makes it a suitable alternative to graphene for energy storage systems.

Several studies have reported the charge storage performance of graphitic carbon nitride. For example, Gonçalves et al. achieved a maximum specific capacitance of 113 F g^–1^ at 0.2 A g^–1^ in a LiClO_4_ electrolyte using urea as a precursor.^9^ Tahir et al. synthesized tubular g-C_3_N_4_ using melamine, which exhibited a specific capacitance of 233 F g^–1^ at 0.2 A g^–1^ in a 6 M KOH electrolyte.^18^ The synthesis of 1D structured graphitic carbon nitride nanofibers by Tahir et al. resulted in a maximum specific capacitance value of 71 F g^–1^ at a current density of 0.5 A g^–1^ in 0.1 M Na_2_SO_4_.^19^ The electrochemical performance of g-C_3_N_4_ composites is also impressive. For example, Zhou et al. synthesized flower-like PANI/g-C_3_N_4_, which showed a high capacitance of 583.4 F g^–1^ at a current density of 1 A g^–1^.^20^ Shi reported a maximum specific capacitance of 505.6 F g^–1^ at 0.5 A g^–1^ for flower-like Ni(OH)_2_/g-C_3_N_4_.^21^

However, the limited knowledge about the local structure of synthesized graphitic carbon nitride limits its viability for use in various applications.^22−24^ For example, functionalization could improve the properties of a material, similar to what has been done with carbon nanotubes and fullerenes. However, the confined states of as-synthesized C_3_N_4_ can limit the functional groups in their interlayers.^12,13,25^ Direct protonation is a feasible method for transforming the thick stacked layers into fine nanolayers and tuning their properties, such as electronic structure in polymers and polymer dendrimers, to enhance proton conductivity and photoluminescence. Zhang et al. proposed a protonation mechanism for g-C_3_N_4_, where the exfoliation process converts the stacked nanosheets into porous nanolayers with high surface area and better ionic conductivity.^25^

In this study, we examine the effect of combining thermal oxidative polymerization and protonation by various acids on the charge storage performance of graphitic carbon nitride. The protonation of C_3_N_4_ by strong mineral acids creates active acid sites that weaken the van der Waals interactions between the interlayers. This study helps explore the mechanisms of different monobasic and dibasic acids for the modification of thermal oxidatively synthesized C_3_N_4_. The counterion exchange potential of the acids, as well as their impact on the surface area of the material, will also be studied. We will investigate the effect of acid treatments, including H_2_SO_4_, HNO_3_, and HCl, on the electrochemical and morphological properties of thermal oxidatively synthesized C_3_N_4_.

## 2 Materials and Methods

### 2.1 Materials

Urea, sulfuric acid (H_2_SO_4_), hydrochloric acid (HCl), and nitric acid (HNO_3_) were purchased from Qualigens. Potassium hydroxide (KOH) and lithium iodide (LiI) was purchased from Molychem. Poly(ethylene oxide) [(PEO) *M*_W_ ∼ 6,00,000] was purchased from Sigma-Aldrich Chemicals. Poly(ethylene glycol) dimethyl ether (PEGDME) was purchased from Tokyo Chemical Industry. Super P carbon and poly(vinyldene fluoride) were purchased from Alfa Aesar. *N*-Methyl-2-pyrrolidone and acetonitrile were purchased from LOBA Chemie. All analytical grade chemicals were used for synthesis without any further purification

### 2.2 Synthesis of Bulk C_3_N_4_

In a typical synthesis, 10 g of urea was transferred to an alumina crucible, which was covered with aluminum foil and placed in a tubular furnace. The temperature was then increased to 550 °C with a heating ramp rate of 3 °C per minute. The crucible was maintained at 550 °C for 2 h, after which the product was referred to as C_3_N_4_–B.

### 2.3 Protonation of Bulk C_3_N_4_

One gram of pristine C_3_N_4_ was added to 100 mL of a 1 M HCl solution and subjected to ultrasonic dispersion for 3 h at room temperature. The protonated C_3_N_4_ was then washed several times with distilled water and dried at 60 °C. In addition to HCl, various other acids such as sulfuric acid (H_2_SO_4_) and nitric acid (HNO_3_) were also used for protonation. The resulting products were named C_3_N_4_–B, C_3_N_4_–H_2_SO_4_, C_3_N_4_–HNO_3_, and C_3_N_4_–HCl, respectively.

### 2.4 PEO/PEGDME/KOH/LiI Gel Electrolyte

Then, 0.48 g of PEO and 0.72 g of PEGDME were added to a 10 mL acetonitrile solution. The mixture was vigorously stirred for 2 h. Finally, 2.24 g of KOH and 0.12 g of LiI dissolved in distilled water were slowly added to the solution under stirring. The solution was continuously stirred until a homogeneous viscous gel was formed. The water/acetonitrile ratio was approximately 90:10.

### 2.5 Characterization

The crystalline phase and purity of the prepared pristine C_3_N_4_ and various acid-treated C_3_N_4_ were studied by X-ray diffraction (XRD) using a PANalytical XPERT-PRO X-ray diffractometer with Cu Kα radiation (λ = 1.5405 A°) at a step angle of 0.02°. The Raman spectra of pristine C_3_N_4_ and C_3_N_4_ treated with various acids were acquired using WITec Alpha-300R with 785 nm laser wavelength. Fourier transform infrared (FTIR) spectroscopy (PerkinElmer) was performed to analyze the molecular vibrations of the prepared pristine C_3_N_4_ and various acid-treated C_3_N_4_ within the wavelength range of 500–4000 cm^–1^. Diffuse reflectance absorption spectroscopy of C_3_N_4_ and various acid-treated C_3_N_4_ was performed by JASCO-V-770. The surface morphologies of the prepared pristine and HCl-treated C_3_N_4_ were acquired by scanning electron microscopy (Carl Zeiss). For transmission electron microscopy (TEM) analysis, the diluted aqueous suspension of C_3_N_4_ was placed on a carbon-coated copper grid and air-dried overnight at room temperature to remove moisture. After drying, the samples were mounted on a high-resolution TEM and imaged at an accelerating voltage of 200 kV (HR-TEM, 2100 Plus, JEOL, Japan). An X-ray photoelectron spectrometer (Omicron Nano Technology, UK) was used to detect the chemical state and atomic percentage of pristine and HCl-treated C_3_N_4_. The Brunauer–Emmett–Teller surface area and pore distribution were analyzed based on N_2_ adsorption–desorption isotherms (Tristar II, Micromeritcs).

### 2.6 Electrochemical Studies

A platinum foil, active-material-coated Ni foam, and a saturated calomel electrode were used as the counter electrode, working electrode, and reference electrode, respectively. Techniques such as cyclic voltammmetry and galvanostatic cycling with potential limitation were used to study the charge storage behavior, and electrochemical impedance spectroscopy was performed to gain deep insights into the electrode–electrolyte interface in the frequency range of 10 kHz to 100 mHz with a current amplitude in the range of 10 mV for pristine C_3_N_4_ (C_3_N_4_-B) and various acid-treated C_3_N_4_ (C_3_N_4_-H_2_SO_4_, C_3_N_4_-HNO_3_, and C_3_N_4_-HCl) in a 6 M KOH solution at a temperature of 23 °C. The experiments were repeated thrice to determine their reproducibility. The mass of the material loading is about 1.5 mg.

Eq 1 is used to calculate the specific capacitance of the material, where *C* denotes the specific capacity of the material; *I* and *t* are the discharge current and time, respectively; and *m* is the mass of the active material.
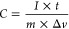
1

### 2.7 Two-Electrode Configurations

The working electrode was prepared by mixing the active material, PVDF, and carbon black super P in the ratio of 80:10:10 to form a slurry in *N*-methyl-2-pyrrolidone (NMP) solvent. The resulting slurry was then coated onto an aluminum foil collector using the doctor-blade technique and dried in an oven at 60 °C. The dried film was rolled into a thin sheet with an optimized thickness and then cut into circular disks with a diameter of 17 mm. HCl-treated C_3_N_4_ was used as both the positive and negative electrodes with a Celgard separator of 0.25 μm thickness in a KOH/LiI gel electrolyte for the two-electrode configuration using a CR2032 setup. Charge and discharge processes were performed at cell voltages of 1.8 and 1.2 V, respectively. Electrochemical impedance spectroscopy was performed in a frequency range of 10 kHz to 100 mHz and current amplitude perturbed signals of about 10 mV. The load of the active material was around 2 mg cm^–2^. The specific capacitance was calculated using the formula given in eq 2, where *C* denotes the specific capacitance of the electrode materials (F g^–1^), *I* and *t* denote the discharge current (mA) and time (s), respectively, *m* is the total mass of the material in both the electrodes, and Δ*v* is the working potential window.
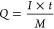
2The performance of the fabricated supercapacitor is related to its energy and power density. The energy density (*E*) and power density (*P*) were calculated using eqs 3 and 4, where *C* denotes the specific capacitance of the device (Fg^–1^), Δ*v* is the potential window (V), and Δ*t* is the discharge time (s).
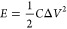
3
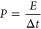
4

## 3 Results and Discussion

**Figure 1:**
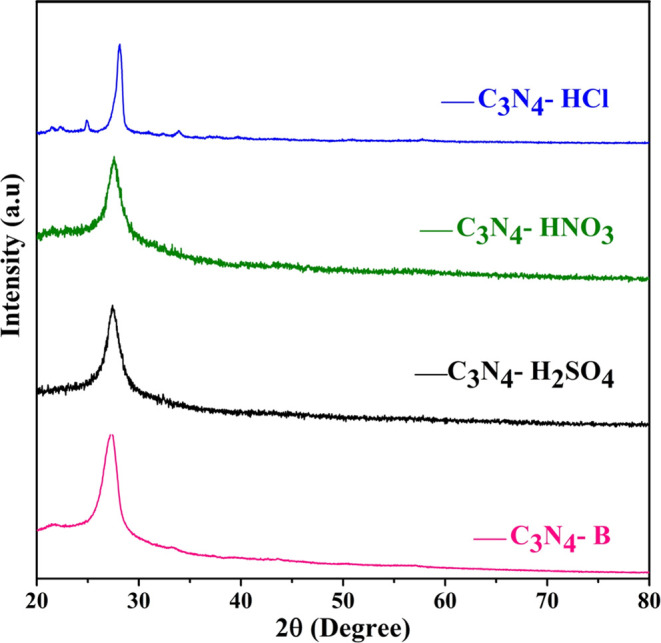
X-ray diffraction patterns of C_3_N_4_-B, C_3_N_4_-H_2_SO_4_, C_3_N_4_-HNO_3_, and C_3_N_4_-HCl.

The X-ray diffraction (XRD) pattern of pristine C_3_N_4_ and the acid-treated C_3_N_4_ (C_3_N_4_–H_2_SO_4_, C_3_N_4_–HNO_3_, and C_3_N_4_–HCl) is shown in Figure 1. It is evident that the characteristic peak (002) observed at an angle of 27.13° corresponds to the carbon nitride structure. The diffracted peak was indexed according to the JCPDS card no. 87-1526. All the acid-treated and pristine samples show similar diffraction peaks except for the HCl-treated sample. This peak is mainly due to the existence of hydrogen bonds that maintain the van der Waals forces between the interlayer stacking of the C–N framework.^26−28^ A significant peak shift and also a drastic reduction in the intensity observed for the acid-treated C_3_N_4_ corroborates the efficient exfoliation of the stacked layers.^29,30^ Regarding the HCl-treated C_3_N_4_, there is a noticeable shift in phase from 27.13 to 28.13° with a significant reduction in the intensity, which implies decreased correlation length by nanostructuring.^31,32^ Additionally, there is a reduction in the peak intensity of the (002) diffracted peak after post-treatment with acids such as H_2_SO_4_, HNO_3_, and HCl, which clearly indicates a lower number of aligned layers and a smaller planar size.^33,34^

**Figure 2:**
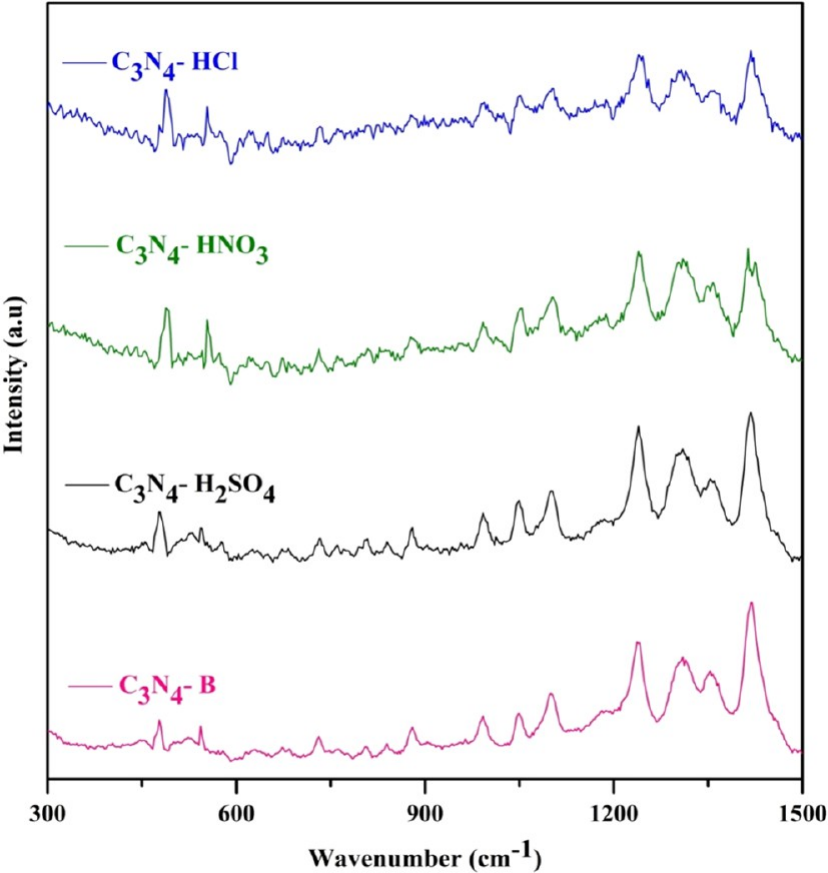
Raman spectra of C_3_N_4_-B, C_3_N_4_-H_2_SO_4_, C_3_N_4_-HNO_3_, and C_3_N_4_-HCl.

Raman spectroscopy was performed to examine the exfoliation of bulk layer C_3_N_4_ into a few layers by various acids. There was more interference from the fluorescence behavior of the material; it was further reduced by decreasing the energy of a laser diode with an excitation wavelength of 785 nm over a long period of time. The depicted Raman spectra are shown in Figure 2. The characteristic strong bands of 1418, 1351, 1310, 1234, 1099, 1048, 545, and 479 cm^–1^ and weak bands of 989, 880, 842, 806, and 729 cm^–1^ were observed for pristine and acid-treated C_3_N_4_. The 989, 880, 842, 806, and 729 cm^–1^ bands diminished after the acid treatment; they are associated with the deformation vibration of CN heterocycles and are usually attributed to layer deformation.^35^ The band at 1234 cm^–1^ corresponds to the bending vibration of the =NH_2_ band, and across all acid-treated C_3_N_4_, a consistent shift toward the higher frequency was observed, which may be due to the quantum confinement of their ultrathickness.^35^ The several bands observed from 1000 to 1500 cm^–1^ were associated with the stretching vibration of C=N and N–H deformation.^36^ There was a significant change in the wavenumber and intensity of the acid-treated C_3_N_4_ compared with the bulk in a similar pattern. The vibration band at 545 cm^–1^ corresponds to the in-plane symmetrical and twisting vibration of the S heptazine ring, while exfoliation through acid does not affect the S heptazine ring in the C–N framework.^35^ The vibration bands at 479 and 545 cm^–1^ are correlated to layer–layer deformation and weak interactions between stacked interlayers. Accordingly, the intensity of the band increases for the pristine and acid-treated C_3_N_4_ for each acid, which clearly depicts the exfoliation of the interlayer. The ratio of *I*_549_/*I*_479_ was found to be increased after acid treatment, with initial values of 0.89 for the pristine material, 0.93 for H_2_SO_4_ treatment, 0.90 for HNO_3_ treatment, and 1.01 for HCl treatment. Decreasing the layers in the CN structure results in a reduction of the conjugation length, possibly attributable to the quantum confinement effect, which induces opposing shifts in conduction and valence bands, which clearly illustrates the exfoliation implying their thickness. This confirms that acid protonation separates the stacked layers efficiently.^37^

**Figure 3:**
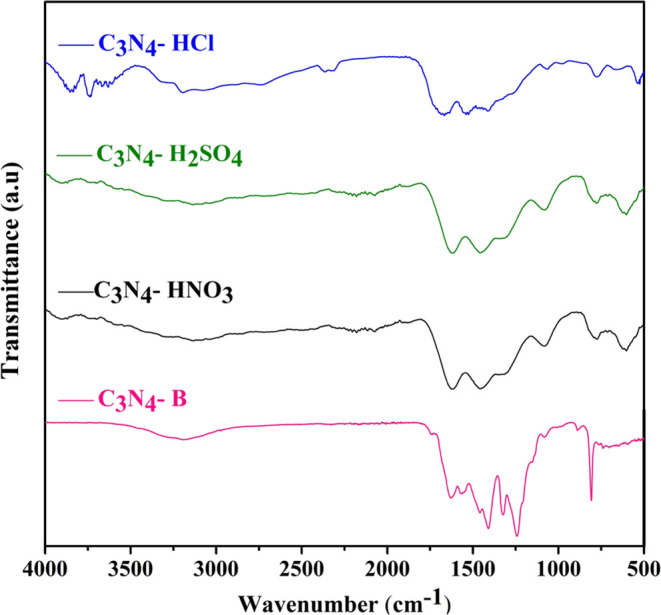
FTIR spectra of C_3_N_4_-B, C_3_N_4_-H_2_SO_4_, C_3_N_4_-HNO_3_, and C_3_N_4_-HCl.

FTIR spectroscopy was performed to explicate the chemical structure of pristine and various acid-treated C_3_N_4_ (Figure 3). The characteristic peak of 810 cm^–1^ observed for the pristine and acid-treated C_3_N_4_ corresponds to the bending vibration of the s-triazine ring.^38^ After the acid treatment, a decreased intensity and a phase shift were observed at a wavenumber of 810 cm^–1^, indicating the structural evolution of the tri-s-triazine ring due to the segregation of the stacked interlayer in the C–N framework.^39,40^ This clearly renders the extended tri-s-triazine unit with an enhanced Π-conjugated system. In addition, a broad absorption band ranging from 3000 to 3500 cm^–1^ is observed for the pristine and acid-treated C_3_N_4_ due to the stretching vibration of the uncondensed primary NH and secondary NH_2_ groups in the CN heterocyclic skeleton.^41^ Various absorption bands observed for the pristine and acid-treated C_3_N_4_ occur in the region of 1200–1640 cm^–1^ and are associated with the stretching vibration of C–N and C=N aromatic heterocyclic repeating units.^42^

**Figure 4:**
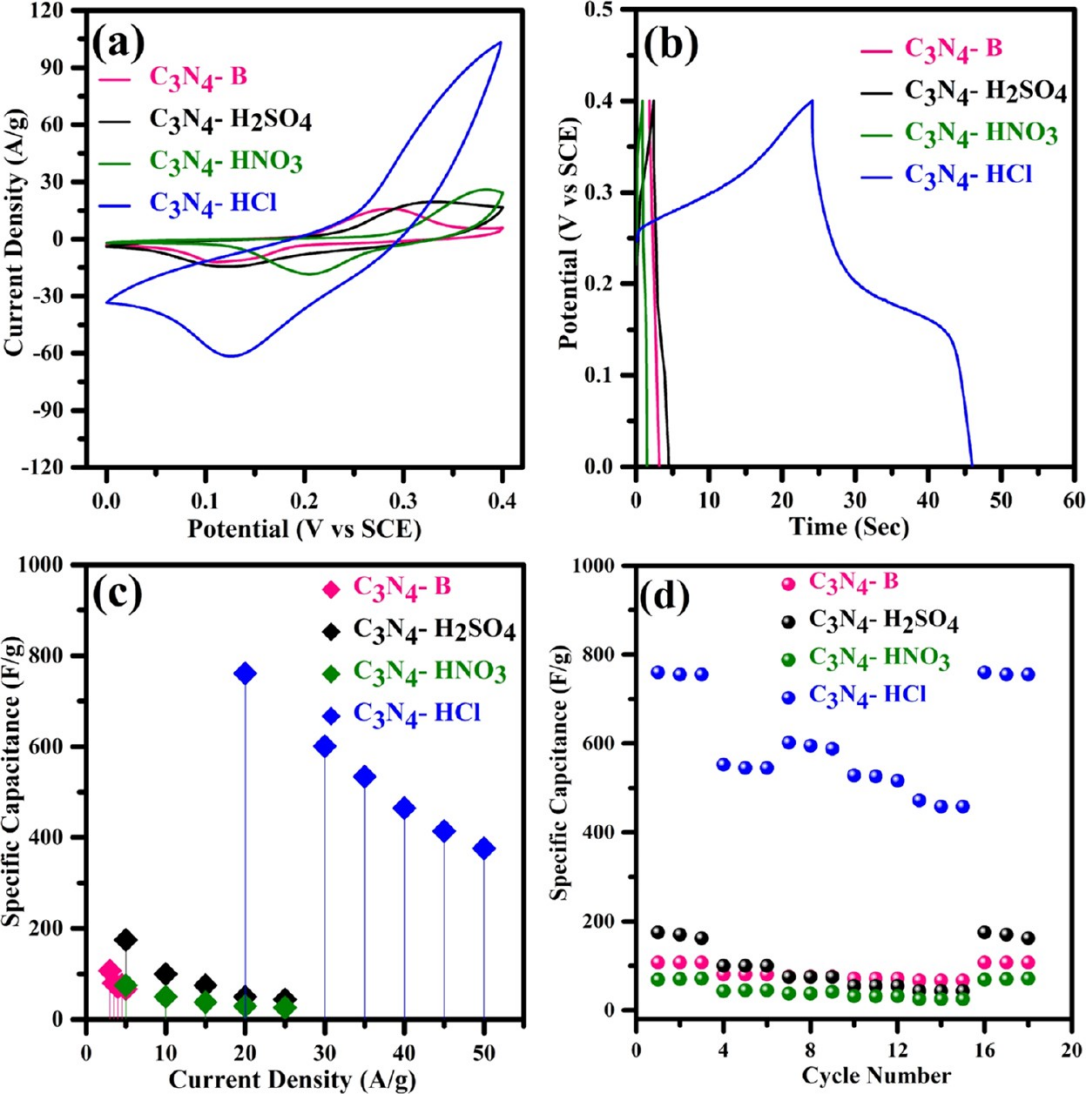
(a) CV profiles of C_3_N_4_-B, C_3_N_4_-H_2_SO_4_, C_3_N_4_-HNO_3_, and C_3_N_4_-HCl at a scan rate of 100 mV s^–1^. (b) Charge–discharge profiles of C_3_N_4_-B, C_3_N_4_-H_2_SO_4_, C_3_N_4_-HNO_3_, and C_3_N_4_-HCl at a current density of 20 A g^–1^. (c) Plot of the specific capacity vs current density. (d) Plot of the specific capacity vs cycle number at various current densities.

The electrochemical performances of the pristine (C_3_N_4_-B) and three acid-treated forms of carbon nitride (C_3_N_4_-H_2_SO_4_, C_3_N_4_-HNO_3_, and C_3_N_4_-HCl) were studied using a three-electrode configuration in 6 M KOH electrolyte. Figure 4a shows the cyclic voltammogram profile of the pristine and acid-treated C_3_N_4_ at a scan rate of 100 mV s^–1^, which highlights the fact that the acid-treated C_3_N_4_ exhibits improved current density compared to the pristine form. This is due to the unique properties of each acid, which dissociate the stacked layers of C_3_N_4_, resulting in the potential release of the base functionalities (−C–N) that have a high content of nitrogen. The sulfuric acid-treated C_3_N_4_ displays redox peaks similar to that of the pristine form with a high current density. In contrast, the nitric acid-treated C_3_N_4_ shows shifted redox peaks. The increased current density signifies an efficient separation of stacked layers. The better performance of the hydrochloric acid-treated C_3_N_4_, despite being a monoprotic acid without OH groups, is due to its ability to enhance the current density and provide more electrochemically active surface area.

Lee^43,44^ devised a technique to discern capacitive elements originating from the surface-adsorbed layer and intercalation reaction. Their assumption was that the diffusion of electrolytic ions from the bulk electrolyte to the electrode surface follows a semi-infinite diffusion length. Thereby, the areal specific capacitance of the material decreases. Consequently, as the scan rates increase, the areal specific capacitance of the material diminishes. The relationship between the areal capacitance and ν^1/2^ and ν^–1/2^ indicates a strong linear correlation, providing significant insights into the overall capacitance of the diffused layer capacitance, electrical double-layer capacitance, and pseudocapacitance.

**Figure 5:**
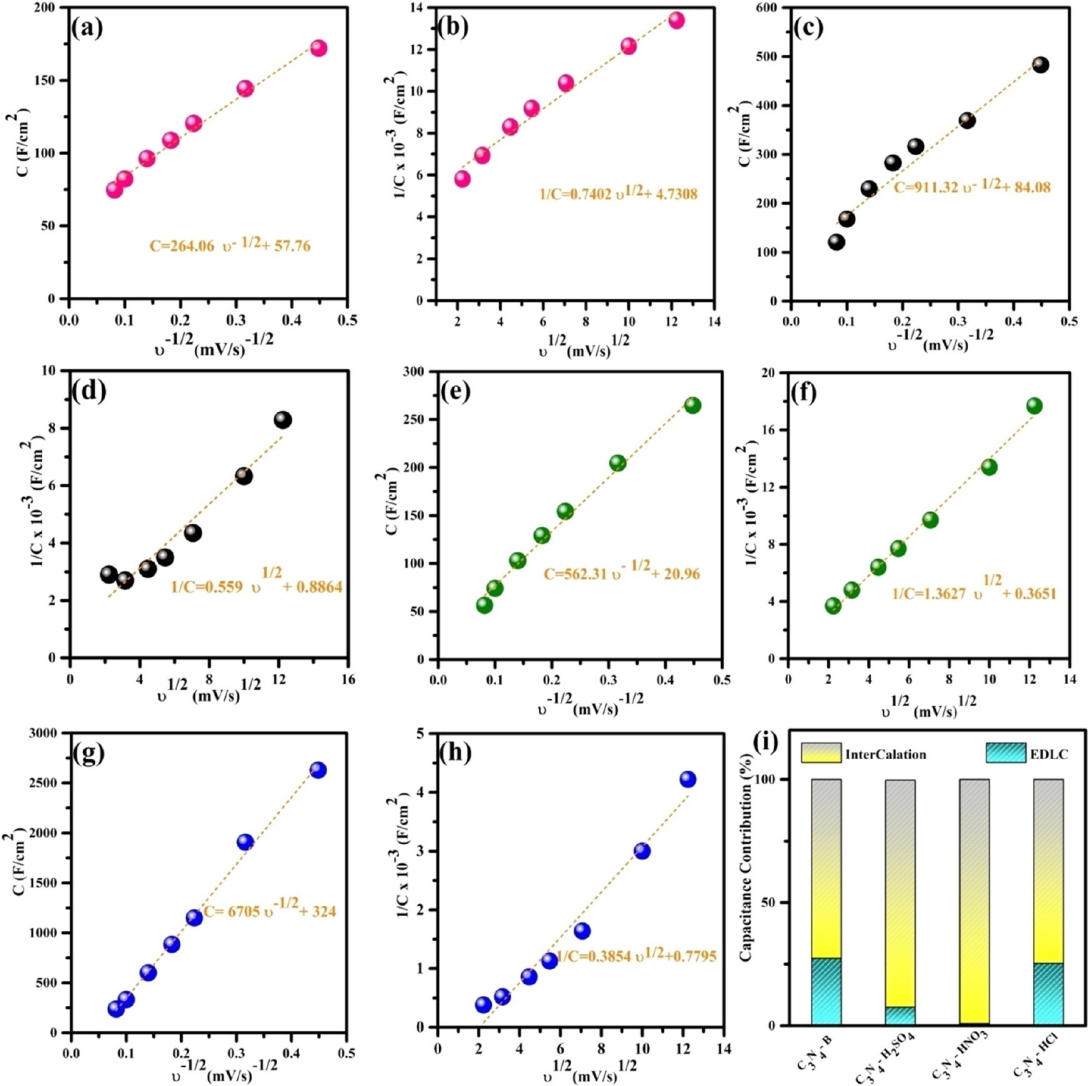
Trasatti analyses of C_3_N_4_-B, C_3_N_4_-H_2_SO_4_, C_3_N_4_-HNO_3_, and C_3_N_4_-HCl. (a, c, e, g) Plots of the areal capacitance vs the reciprocal of the square root of the scan rate (ν^–1/2^) for C_3_N_4_-B, C_3_N_4_-H_2_SO_4_, C_3_N_4_-HNO_3_, and C_3_N_4_-HCl, respectively. (b, d, f, h) Plots of the areal capacitance vs the square root of the scan rate (ν^1/2^) for C_3_N_4_-B, C_3_N_4_-H_2_SO_4_, C_3_N_4_-HNO_3_, and C_3_N_4_-HCl, respectively. (i) Overall capacitance contributions of C_3_N_4_-B, C_3_N_4_-H_2_SO_4_, C_3_N_4_-HNO_3_, and C_3_N_4_-HCl.

Let us consider a scenario where the electrode potential is at zero charge or the scan rate is low (5 mV s^–1^), and sufficient time is provided for the electrolyte ions to partake in the electrochemical reaction. Under these conditions, the overall capacitance of the electrode is predominantly governed by the diffuse layer of electrolyte ions. This can be determined by extrapolating the areal capacitance against the reciprocal square root of the scan rate (ν^–1/2^) (as shown in Figure 5a,c,e,g). Conversely, when assuming infinite diffusion of the electrolyte ions, the charge is stored through adsorption on the electrode surface. The electrical double-layer capacitance depends on the resistance of the electrode materials and can be obtained by extrapolating the areal capacitance against the square root of the scan rate (ν^1/2^), as depicted in Figure 5b,d,f,h. The disparity between the total capacitance and the electrical double-layer capacitance yields a pseudocapacitance arising from the redox reaction between the inner electrode surface and the adsorbed active ionic species.^45,46^ The linear behavior in Figure 5a–h at high scan rates indicates a reduced occurrence of irreversible redox reactions at the electrode material.^46^

The aforementioned Trasatti analysis aids in a quantitative understanding that the overall charge storage contribution of the pristine and various acid-treated C_3_N_4_ stems from the relative proportion of pseudocapacitance and double-layer capacitance (Table S1). Figure 5i shows that the total capacitance of pristine C_3_N_4_ stems from 27.4% electrical double-layer capacitance and 72.6% pseudocapacitance, the total capacitance of C_3_N_4_-H_2_SO_4_ consists of 7.45% electrical double-layer capacitance and 92.5% pseudocapacitance, the overall capacitance of C_3_N_4_-HNO_3_ consists of 0.8% electrical double-layer capacitance and 99.2% pseudocapacitance, and the overall capacitance of C_3_N_4_-HCl is comprised of 25.3% electrical double-layer capacitance and 74.7% pseudocapacitance. The sulfuric acid- and nitric acid-treated C_3_N_4_ showed higher charge storage contribution than pristine C_3_N_4_ due to their efficient delamination of the stacked interlayer due to their increased electron-rich nitrogen moieties contributing directly to their intercalation behavior. However, the pristine and HCl-treated C_3_N_4_ showed synergistically relative proportions of the electrical double-layer and pseudocapacitive behavior. Overall, the HCl-treated C_3_N_4_ synergistically interplayed the effects of the electrical double-layer and pseudocapacitive behaviors, and the effective segregation of the stacked interlayer with their strong electronegativity directly contributes to their capacitance and rate capability.

Figure 4b shows the galvanostatic charge–discharge profile of the pristine and acid-treated C_3_N_4_ at a current density of 20 A g^–1^, along with the calculated specific capacitance values. The calculated specific capacitances of the pristine and acid-treated C_3_N_4_ at various current densities and cycle numbers are shown in Figure 4c,d and Table S2. The maximum specific capacities of the pristine C_3_N_4_-B and acid-treated forms (C_3_N_4_-H_2_SO_4_, C_3_N_4_–HNO_3_, and C_3_N_4_-HCl) are approximately 107, 175, 75, and 761 F g^–1^, respectively. The hydrochloric acid-treated C_3_N_4_ exhibits a 7-fold increase in specific capacity compared to the pristine form and other acids. The can be attributed to the enhanced surface area resulting from the transformation of the bulk layer into nitrogen-rich nanosheets (−C–N). This structural modification facilitates efficient charge accumulation between each layer, contributing to the increased capacitance. Moreover, the Trasatti analysis reveals a synergistic interplay between the effective double-layer formation and redox reactions taking place at the interfaces between the electrode and the electrolyte.

**Figure 6:**
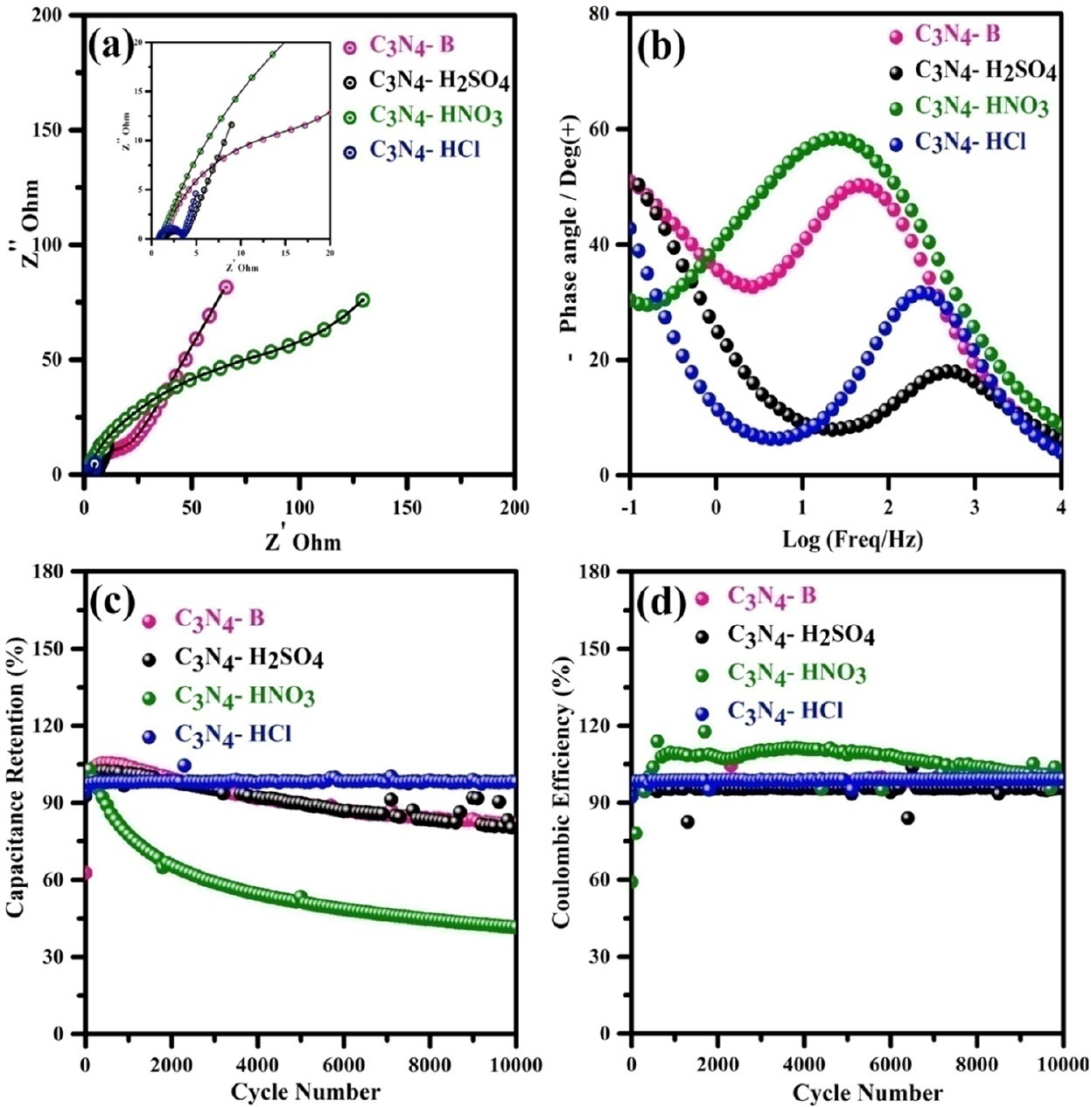
(a) Nyquist plots of C_3_N_4_-B, C_3_N_4_-H_2_SO_4_, C_3_N_4_-HNO_3_, and C_3_N_4_-HCl. (b) Bode phase angle vs frequency plots of C_3_N_4_-B, C_3_N_4_-H_2_SO_4_, C_3_N_4_-HNO_3_, and C_3_N_4_-HCl. (c) Electrochemical cyclic stability as a function of the cycle number for C_3_N_4_-B, C_3_N_4_-H_2_SO_4_, C_3_N_4_-HNO_3_, and C_3_N_4_-HCl. (d) Coulombic efficiency as a function of the cycle number for C_3_N_4_-B, C_3_N_4_-H_2_SO_4_, C_3_N_4_-HNO_3_, and C_3_N_4_-HCl. Note that the sphere represents experimental data, and the straight line represents the fitted data presented in the Nyquist plot of (a).

Figure 6c,d illustrates the capacitance retention and Coulombic efficiency with respect to the number of cycles for the pristine (C_3_N_4_-B) and acid-treated (C_3_N_4_-H_2_SO_4_, C_3_N_4_–HNO_3_, and C_3_N_4_-HCl) forms. The pristine C_3_N_4_ exhibits a cyclic stability of 95% with high reversibility of charged ions to its initial capacitance. The good rate capability of the material aligns well with the Trasatti analysis, which emphasizes the fact that 27.4% of the electrical double layer leads to the effective construction of charged ions, whereas 72.6% is associated with surface redox reactions. Even though the nitric acid and sulfuric acid treatment of C_3_N_4_ leads to a higher capacitance compared to the pristine form, it also results in a lower rate capability. This discrepancy arises from the delamination of the layered sheets during the treatment process. Although delamination increases the surface area and promotes capacitance, it simultaneously leads to the breakdown of the layer due to the reduction of the active species during the cycling mechanism. This agrees well with the Trasatti analysis, which emphasizes that both sulfuric and nitric acid are ineffective in charged ion construction, with a high pseudocapacitive behavior leading to the breakdown of the layer. The hydrochloric acid-treated C_3_N_4_ exhibits exceptional cyclic stability of 100% over 10 000 cycles without sacrificing its Coulombic efficiency. This structural modification enables improved charge accumulation between individual layers, thereby enhancing the overall capacitance. Furthermore, the Trasatti analysis elucidates a synergistic interplay between the formation of an effective double layer and the occurrence of redox reactions at the electrode–electrolyte interfaces. This interplay further enhances the rate capability compared to the pristine and other acid-treated C_3_N_4_.

The Nyquist plots (Figure 6a) of the pristine and various acid-treated C_3_N_4_ were used to study the interface reaction between the electrode and the electrolyte, and an equivalent circuit consisting of a resistor and a capacitor was fitted. The obtained impedance spectra were fitted with a suitable equivalent circuit, the fitted elements of which are shown in Figure S1 and Table S3. The values of *R*_1_ for all acid-treated and pristine C_3_N_4_ were low, indicating good material conductivity. The high values of *Q*_2_ and *R*_2_ for the HCl-treated C_3_N_4_ indicated that the storage contribution mainly originated from the high mass charge transfer due to the strong electronegativity of the nitrogen, while the electrode surface interacts with the electrolytic ion. After cycling, the value of *R*_2_ increased for the pristine, sulfuric acid-treated, and hydrochloric acid-treated C_3_N_4_, while it decreased for nitric acid, emphasizing that the reduction of electrochemical species upon cycling indicated a lower cyclic stability for nitric acid than for the other materials. The high value of *Q*_3_ for pristine and other acid-treated materials, such as sulfuric acid and nitric acid, implied a charge storage contribution from the pseudocapacitive behavior due to the diffusion of electrolytic ions into the electrode.

The Bode phase plot of pristine and various acid-treated C_3_N_4_ (Figure 6b) showed a strong capacitive nature in the middle-frequency region for all materials. The hydrochloric acid-treated C_3_N_4_ articulated at an angle of 45° in the low-frequency region, indicating the diffusion of electrolytic ions with high mass charge transfer due to its improved nitrogen content. In contrast, sulfuric acid and pristine C_3_N_4_ articulated at an angle of 52°, but had lower storage contribution than hydrochloric acid. Nitric acid-treated C_3_N_4_ had a low-frequency region at an angle of 29°, with locked electrolytic ions, indicating a lower storage capacitance. The detailed electrochemical performance of pristine and acid-treated C_3_N_4_ is provided in the Supporting Information (Figures S2–S5). The absorption behavior of pristine and acid-treated C_3_N_4_ is provided in the Supporting Information (Figure S6). Likewise, the charge storage behavior of hydrochloric acid-treated C_3_N_4_ was reproduced and is shown in Figure 11. The reproducibility was determined at different periods in a three-electrode configuration with the optimized mass loading. Figure 11a,b displays the galvanostatic charge–discharge profile and their corresponding specific capacitance values. The charge–discharge behavioral pattern was similar where the discharge time varies. The average value from the repeatability measurement with maxima and minima is represented in Figure 11b.

**Figure 7:**
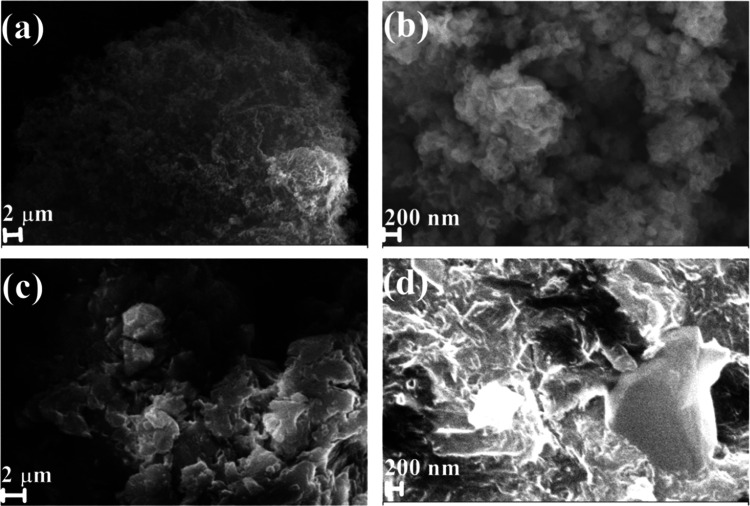
SEM images of C_3_N_4_-B at scales of (a) 2 μm and (b) 200 nm. SEM images of C_3_N_4_–HCl at scales of (c) 2 μm and (d) 200 nm.

The surface morphology of the prepared pristine C_3_N_4_ and hydrochloric acid-treated C_3_N_4_ is shown in Figure 7. The thermal polymerization of urea effectively constructed a sponge-like stacked C–N framework. Figure 7a,b shows the higher and lower magnifications of pristine C_3_N_4_, where the stacked lamellar structure with the interlinked C–N framework provides higher accessibility to electrolytic ions. The hydrochloric acid-protonated C_3_N_4_ efficiently segregated the stacked piled-up layer into a few layers. The SEM image (Figure 7c,d) clearly shows that the exfoliated layer measures a few nanometers.

**Figure 8:**
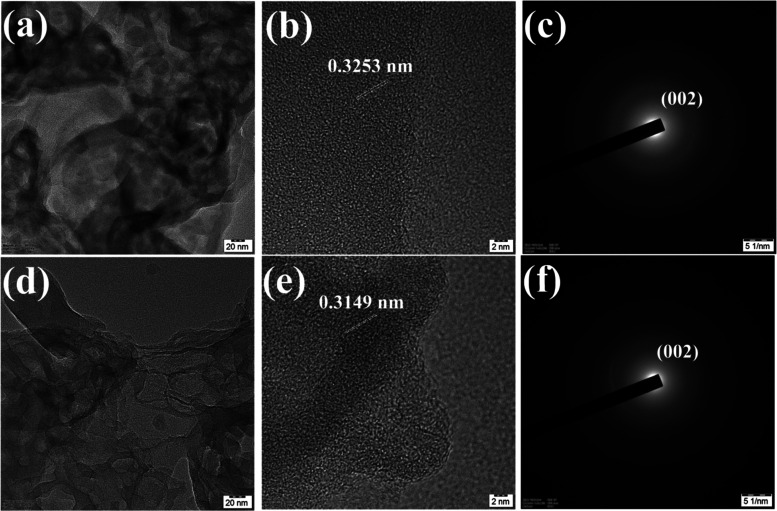
HRTEM images of (a) C_3_N_4_-B and (d) C_3_N_4_-HCl at 20 nm scale. SAED patterns of (b, c) C_3_N_4_-B and (e, f) C_3_N_4_-HCl.

The SEM images of the prepared pristine C_3_N_4_ and hydrochloric acid-treated C_3_N_4_ show a very rough and deteriorated irregular graphene sheet-like morphology.^4^ The fine structures of pristine C_3_N_4_ and hydrochloric acid-treated C_3_N_4_ were determined by high-resolution transmission electron microscopy. In the TEM image (Figure 8) at a 20 nm scale bar, the layered sheet-like morphology is clearly evident. In Figure 8a, it is obvious that pristine C_3_N_4_ shows a thick nanoporous multilayered morphology, whereas protonation segregates the stacked interlayer resulting in a multilayered, thin nanoporous structure (Figure 8d). At a scale bar of 50 nm, the black and dark spots indicate the presence of nanopores (Figure S7). The SAED pattern of pristine C_3_N_4_ shows the lattice fringes of multilayered sheets with an interplanar *d* spacing of 0.3253 nm, corresponding to the 002 plane of the graphitic structure (Figure 8c). The hydrochloric acid-treated C_3_N_4_ reveals lattice fringes of 0.3149 nm, which correspond to the 002 graphitic plane (Figure 8f). The elemental composition in terms of the weight and atomic percentage of pristine C_3_N_4_ and hydrochloric acid-treated C_3_N_4_ obtained from TEM analyses are presented in Table S4 and Figure S8. It is apparent that the atomic percentage of nitrogen in hydrochloric acid-treated C_3_N_4_ increased by a value of 1.54. Thus, the protonation process increases the nitrogen content in C_3_N_4_ directly imparting accessibility to electron-rich nitrogen sites, which significantly enhances their adsorption properties due to their strong electronegative nature. Accordingly, the acid treatment of C_3_N_4_ breaks the weak van der Waals interaction between their layers due to the unconfined proton released from the hydrochloric acid. The separated ultrathin layers improve the nitrogen content in C_3_N_4_ by reducing the carbon content of the C–N framework, which provides more activation sites due to their electron-enriched nitrogen moieties.

**Figure 9:**
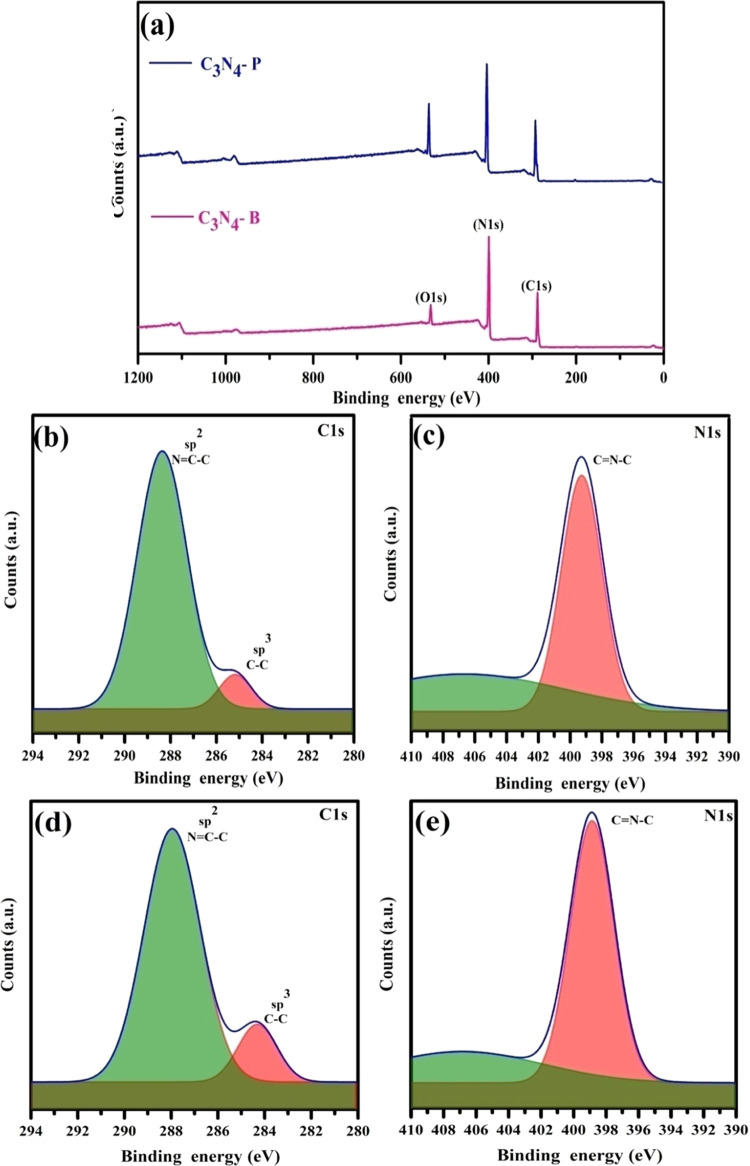
HR-XPS spectra of C_3_N_4_-B and C_3_N_4_-HCl. (a) Full scan spectra of C_3_N_4_-B and C_3_N_4_-HCl. (b, d) C 1s region of C_3_N_4_-B and C_3_N_4_-HCl. (c, e) N 1s region of C_3_N_4_-B and C_3_N_4_-HCl.

XPS was performed to determine the surface chemical composition of pristine C_3_N_4_ and hydrochloric acid-treated C_3_N_4_ (Figure 9). The XPS survey spectra mainly comprised carbon (C 1s), nitrogen (N 1s), and oxygen (O 1s). The high-resolution XPS spectra of the C 1s region acquired from pristine C_3_N_4_ were deconvoluted into the two binding energies of 288.35 and 285.06 eV, corresponding to the sp^2^ and sp^3^ hybridization of carbon, respectively (Figure 9b,d). However, hydrochloric acid-treated C_3_N_4_ after deconvolution exhibited two binding energy peaks at 287.9 and 284.41 eV, assigned to the sp^2^ and sp^3^ hybridization of carbon, respectively. The sp^3^ peak arises due to the C=C carbon originating from the adventitious carbons present in C_3_N_4_. The stronger sp^2^ peak appeared due to the N=C–C aromatic carbon in the layered C_3_N_4_ framework. The N_1s_ region of pristine C_3_N_4_ is deconvoluted into two binding energies of 399.2 eV (C=N–C) and 404.3 eV (–N=N–) in the aromatic ring structure (Figure 9c). The deconvolution of hydrochloric acid-treated C_3_N_4_ is attributed to two binding energies of 398.2 eV (C=N–C) and 405.04 eV (–N=N–) in the aromatic ring structure (Figure 9e). The peak at 404.3 eV further confirms the graphitic stacking of the CN layer. The intensity of the sp^2^- and sp^3^-hybridized peak C=C drastically increased after the HCl treatment. The nonexistence of the peak at 400 eV is due to the uncondensed −NH_2_- group and bridging C–N=N–C atoms.^47^ The elemental analysis was performed to obtain the atomic percentage by excluding O 1s. The percentage of C/N for the pristine C_3_N_4_ results in a C_B_/N_B_ of 35.30:64.70, whereas the hydrochloric acid-treated C_3_N_4_ shows a C_P_/N_P_ of 34.27:65.73. It is apparent that the atomic percentage of nitrogen in hydrochloric acid-treated C_3_N_4_ increased by a value of 1.03 by reducing the carbon content (Table S5). The adsorbed OH group and adventitious carbon originate from the O 1s region^47^ (Figure S9). The separated layers improve their nitrogen content in C_3_N_4_ by reducing the carbon content of the C–N framework due to their unconfined proton release during the protonation process under ultrasonication. The nitrogen-rich C_3_N_4_ obtained by the facile and scalable synthesis is validated by all of these XPS results.

**Figure 10:**
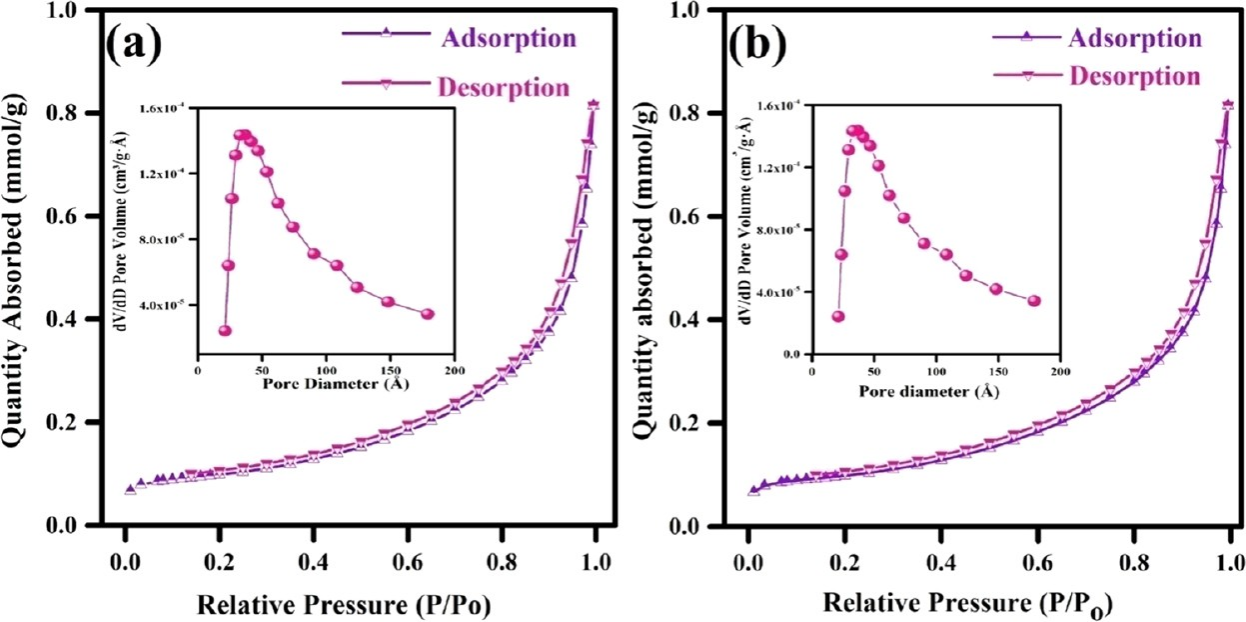
N_2_ adsorption–desorption isotherms of (a) C_3_N_4_-B and (b) C_3_N_4_-HCl (insets show the pore diameter distribution profiles).

**Figure 11:**
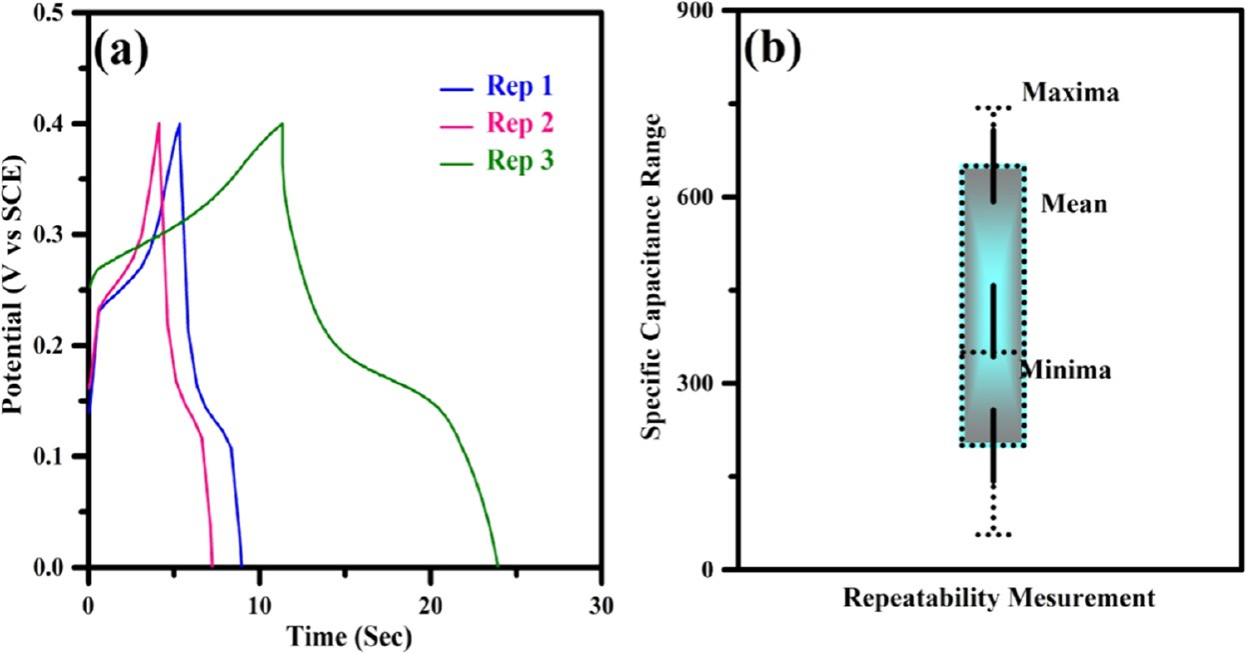
Reproducibility measurement of C_3_N_4_-HCl. (a) Charge–discharge profile of C_3_N_4_-HCl at the current density of 20 A g^–1^. (b) Plot of specific capacity vs repeatability of C_3_N_4_-HCl.

The nitrogen adsorption and desorption isotherms of pristine and hydrochloric acid-treated C_3_N_4_ are represented in Figure 10. A high surface area of about 7.511 m^2^ g^–1^ was obtained for the acid-treated C_3_N_4_ due to the exfoliation of the stacked interlayers. In contrast, pristine g-C_3_N_4_ had a surface area of 1.0319 m^2^ g^–1^. According to the BJH pore size distribution, the pore size diameter of pristine C_3_N_4_ was found to be 32 Å, while the diameter of the hydrochloric acid-treated C_3_N_4_ was about 38 Å. The enhanced surface area and pore size were mainly attributed to the etching of the stacked interlayer into several nanolayers. Hence, the enhanced surface area is not only the main factor that strongly influences their charge storage contribution but also their physical and chemical properties. In conclusion, the increased nitrogen content in C_3_N_4_ hydrochloric acid treatment showed the highest storage capacitance due to the efficient exfoliation of the stacked carbon nitride with electron-rich nitrogen moieties in the C–N framework. A symmetric supercapacitor was constructed to extend its energy storage applications (Figure 11).

### 3.1 Symmetric Supercapacitor

**Figure 12:**
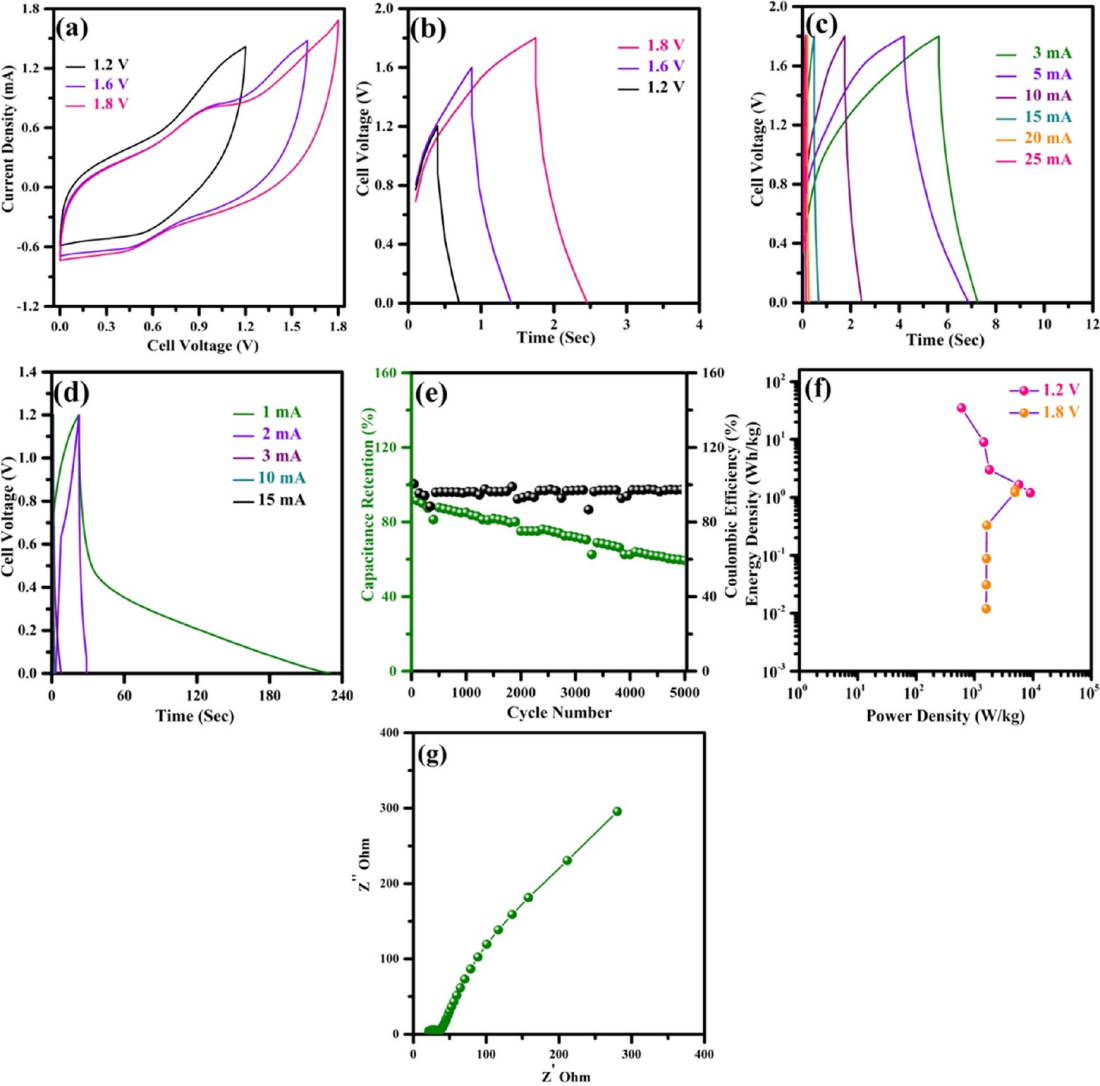
Electrochemical performance of the fabricated symmetric HCl-treated C_3_N_4_ with the PEO/PEGDME/KOH/LiI-based gel electrolyte. (a) CV profile of the symmetric device in various voltage windows at a scan rate of 100 mV s^–1^. (b) Charge–discharge profile of the device in various voltage windows at a current density of 5 A g^–1^. (c) Charge–discharge profile of the symmetric device at various current densities at 1.8 V potential window. (d) Charge–discharge profile of the symmetric device at various current densities at 1.2 V potential window. (e) Electrochemical cyclic stability and Coulombic efficiency as a function of the cycle number of the fabricated symmetric device. (f) Ragone plot of the fabricated symmetric device at 1.2 and 1.8 voltage window. (g) Nyquist plot of the fabricated symmetric device.

A symmetric supercapacitor was fabricated using hydrochloric acid-treated C_3_N_4_ as both the positive and negative electrodes in a KOH gel-based electrolyte with LiI as the electrolyte additive. The cell voltage of the device could charge from 1.2 to 1.8 V. Figure 12a,b displays the cyclic voltammogram and charge–discharge profile of the symmetric device at various voltage windows. The cyclic voltammogram profile suggests that the adsorption/desorption of K^+^OH^–^ occurs due to the supporting electrolyte LiI, leading to the deeper penetration of ions at the electrode surface with some redox behavior noted at all operating voltage windows. There is a very small voltage drop observed in the cell, which emphasizes the excellent electrochemical reversibility of the charged ions. Figure 12g represents the Nyquist representation of the symmetric device. The device exhibits low ESR resistance, indicating good electrical contact between the current collector and the electrolyte. The parameters, including capacitance, energy density, and power density, were calculated using standard equations from the symmetric device.^48−50^ The calculated specific capacitance, energy density (*E*), and power density (*P*) of the fabricated device from the charge–discharge analysis are presented in Table S6.

Initially, the cell was evaluated by charging to 1.8 V at various charging and discharging currents (Figure 12c). It exhibited EDLC behavior with a very low voltage drop at high charging and discharging currents. The maximum specific capacitance of the fabricated device was calculated as 3 F g^–1^ at a current density of 3 A g^–1^. Despite charging the material to 1.8 V, the device showed low values of capacitance, energy, and power density. It achieves an energy density and power density of 1.35 Wh kg^–1^ and 4.9 kW kg^–1^, respectively, at a current density of 3 A g^–1^. Therefore, the operational voltage window was decreased to 1.2 V, which led to an increase in the energy density. Figure 12d displays the charge–discharge studies of 1.2 V at various charging and discharging currents. It shows pseudocapacitive behavior, and the maximum specific capacitance of the fabricated device was calculated as 175 F g^–1^ at a current density of 1 A g^–1^. The Ragone plot of the fabricated symmetric device at 1.2 and 1.8 voltage window is shown in Figure 12f. The device achieved a significant energy density and power density of 35 Wh kg^–1^ and 600 W kg^–1^, respectively, at a current density of 1 A g^–1^. The electrochemical stability of the fabricated device was evaluated for 5000 cycles at a current density of 10 A g^–1^, showing 60% capacitance retention without compromising its Coulombic efficiency (Figure 12e). The significant improvement at an operating voltage of 1.2 V was due to the synergistic effect of the impregnation of the KOH electrolyte and the additive LiI, which aided the redox and EDLC at the electrodes.

The major problems addressed in this supercapacitor are as follows. (i) The pseudocapacitive material delivers a higher capacitance due to the faradic reaction, but it still suffers from cyclic instability due to the reduction of electrochemical active species. (ii) The EDLC material usually possesses a lower specific capacitance due to surface-adsorbed ions and has excellent cyclic stability.^51−54^ These problems were addressed by our findings, where the layered C_3_N_4_ was activated through acid exfoliation. Based on their charge storage behavior, it was discerned that the activation of nitrogen moieties in the C–N framework through the acid exfoliation results in a higher capacitance with excellent cyclic stability compared to studies based on carbon and carbon nitride.^48,51,52,55−57^ A comparison of the performance of carbon nitride in terms of its capacitance, cyclic stability, and energy density is presented in Table 1. The symmetric capacitor of C_3_N_4_ shows an energy density value as high as 35–1.2 Wh kg^–1^ and a power density value as high as 600–9K W kg^–1^, which is higher than those of previously reported C_3_N_4_ supercapacitors.^48,55^ Overall, on the basis of their cost effectiveness, high capacitance with excellent cyclic stability, and processing route, the hydrochloric acid-activated C_3_N_4_ will be a promising metal and carbon-free energy storage material in the near future for sustainable energy development.

**Table 1:** Performance Comparison of Carbon Nitride in Terms of its Capacitance, Cyclic Stability, and Energy Density

Performance Comparison of Carbon Nitride-Based Supercapacitors					
material	capacitance (F g^–1^)/current density (A g^–1^)	capacitance retention-cycle number	energy density (Wh kg^–1^)	operating voltage/electrolyte	ref
g-C_3_N_4_ nanofiber	263.75/1	93.2%–2000	N/A	–0.1 to 0.7 V/0.1 M Na_2_SO_4_	(19)
tubular g-C_3_N_4_	233/0.2	90%–1000	N/A	–0.2 to 1.0 V/6 M KOH	(18)
flower-like Ni(OH)_2_@g-C_3_N_4_	505.6/0.5	71.5%–1000	17.46	0–0.4 V/6 M KOH	(21)
g-C_3_N_4_/Cu-AL LDH	714/0.5	82%–10 000	N/A	0–0.35 V/6 M KOH	(58)
Ni(OH)_2_@g-C3N4 honeycomb structure	51/0.5^a^	72%–8000	43.1	0–1.2 V/6 M KOH	(59)
C-g-C_3_N_4_/GO	379.7/0.5	85%–10 000	52.7	–0.5 to 0.5 V/6 M KOH	(13)
g-C_3_N_4_/GO	265.6/1	94%–5000	14.93	–0.8 to 0 V/2 M KOH	(60)
performance comparison of carbon-based supercapacitors					
carbon nanotube (functionalized by −OH and −COOH)	3199/5	70%–300	N/A	0–1 V/0.075 M hydroquinone (HQ) into 1 M H_2_SO_4_ aqueous	(61)
PEDOT/MWCNT	79/0.5^a^	85%–1000	11.3	0–1 V 1 M LiClO_4_	(62)
rGO	348/0.2	120%–3000	N/A	–0.2 to 0.8 V/1-butyl-3- methylimidazolium hexafluorophosphate (BMIPF_6_)	(63)
rGO hydrogel	220/1	92%–2000	5.7	0–1 V/5 M KOH	(64)
activated carbon	225/1	100%–5000	72.2	0–2 V 1 M TBAPF_6_	(65)
activated carbon	24/0.25	100–10 000	N/A	0–0.8 V/1 M H_2_SO_4_	(66)

This Work				
material	capacitance (F g^–1^)/current density (A g^–1^)	capacitance retention-cycle number	energy density (Wh kg^–1^)	operating voltage/electrolyte
three-electrode configuration	761/20	105%–10 000	N/A	0–0.4 V/6 M KOH
two-electrode configuration	175/1	60%–5000	35	0–1.2 V/PEO/PEGDME/KOH/LiI gel-based electrolyte

a Two-Electrode Configuration.

## 4 Conclusions

The development of a low-cost and efficient strategy has been reported to exfoliate the stacked layers of C_3_N_4_. The crucial factor in this process is the protonation, which increases the reaction sites and improves their electron-rich nitrogen moieties by reducing the carbon content, allowing for improved ionic diffusion into the electrode due to their strong electronegativity. The hydrochloric acid-treated C_3_N_4_ showed a specific capacitance of 761 F g^–1^ at a current density of 20 A g^–1^, which is 7-fold higher than that of other acids in a three-electrode configuration. Moreover, it showed excellent cyclic stability even after 10 000 cycles without any compromise in the Coulombic efficiency. The symmetric supercapacitor was constructed using a KOH/LiI gel-based electrolyte, and it demonstrated a maximum specific capacitance of 175 F g^–1^ at a current density of 1 A g^–1^. Furthermore, the device displayed significant power and energy densities of 600 W kg^–1^ and 35 Wh kg^–1^, respectively. It showed a superior cyclic retention of around 60% even after 5000 cycles with excellent Coulombic efficiency. Overall, the proposed strategy by activating carbon nitride through a protonation process to obtain the metal-free sustainable material and its practical viability paves the way to replacing existing carbonaceous materials.

## Supporting Information Available

Equivalent circuit diagram (Figure S1); high mass charge transfer (S2) electrochemical analysis of pristine C_3_N_4_ and various acid-treated C_3_N_4_ (Figures S2–S5); UV-DRS spectra of pristine C_3_N_4_ and various acid-treated C_3_N_4_ (Figure S6); HRTEM images of (a) C_3_N_4_-B and (b) C_3_N_4_-HCl (Figure S7); elemental compositions of (a) C_3_N_4_-B and (b) C_3_N_4_-HCl obtained through TEM analysis (Figure S8); O 1s regions of C_3_N_4_-B and C_3_N_4_-HCl (Figure S9); cyclic voltammogram profile of bare Ni foam in 6 M KOH solution (Figure S10); X-ray diffraction pattern of pristine C_3_N_4_ and various acid-treated C_3_N_4_ (Figure S11) (PDF)
