# Supplementary material for: Protonated C3N4 Nanosheets for Enhanced Energy Storage in Symmetric Supercapacitors through Hydrochloric Acid Treatment
Source: ACS Omega. 2024 Feb 28;9(10):11273–87. doi: 10.1021/acsomega.3c06747 (PMC10938317; doi:10.1021/acsomega.3c06747)
Supplement: Supplementary file 1 — ao3c06747_si_001.pdf [file ao3c06747_si_001.pdf]

Protonated C_3N_4 Nanosheet for Enhanced Energy Storage in Symmetry Supercapacitors through Hydrochloric Acid Treatment

Mahalakshmi Subbiah[1,2], Annalakshmi Mariappan[2], Anandhakumar Sundaramurthy,[3] Venkatachalam Sabarinathan [1,4], Rajasekaran Thanjavur Renganathan[1], Nishakavya Saravanan[5], Sudhagar Pitchaimuthu[6*], Nagarajan Srinivasan[2*]

1. *Department of Renewable Energy Science, Manonmaniam Sundaranar University, Tirunelveli, India*

2. *Laboratory of Electrochemical Interfaces, Department of Chemistry, Manonmaniam Sundaranar University, Tirunelveli, India.*

3. *Biomaterials Research Laboratory, Department of Chemical Engineering, SRM Institute of Science and Technology, Kattankulathur-603203, Tamil Nadu, India*

4. *Department of Physics, Manonmaniam Sundaranar University, Tirunelveli, India*

5. *Department of Physics and Nanotechnology, SRM Institute of Science and Technology, Kattankulathur-603203, Tamil Nadu, India*

6. *Research Centre for Carbon Solutions (RCCS), Institute of Mechanical, Processing and Energy Engineering, School of Engineering and Physical Sciences, Heriot-Watt University, United Kingdom.*

* Corresponding authors

S.Pitchaimuthu@hw.ac.uk (SP), snagarajan@msuniv.ac.in (SN)

**Material**	**Pesudocapacitance (%)**	**EDLC (%)**
**C_3N_4 – B**	72.66	27.33
**C_3N_4 – H_2SO_4**	92.5	14.55
**C_3N_4 – HNO_3**	99.2	0.8
**C_3N_4 – HCl**	74.7	25.3

Table S1. The calculated pesudocapcitance (%) and EDLC (%) of C_3N_4- B, C_3N_4- H_2SO_4, C_3N_4- HNO_3, and C_3N_4- HCl obtained from tarasatti analysis.

	Current Density (A/g)	3	3.5	4	4.5	5	10	15	20	25	30	40	45	50
Specific Capacitance	C_3N_4 – B (F/g)	107	80	71	76	67	----	15	----	----	----	----	----	----
	C_3N_4-H_2SO_4 (F/g)	----	-----	----	----	175	100	75	50	44	----	----	----	----
	C_3N_4- HNO_3 (F/g)	----	----	----	----	75	50	38	29	26	----	----	----	----
	C_3N_4 – HCl (F/g)	----	----	----	----	----	----	----	761	601	534	465	414	376

Table S2. The calculated specific capacitance of C_3N_4- B, C_3N_4- H_2SO_4, C_3N_4- HNO_3, and C_3N_4- HCl.

S1. Electrochemical behaviour

Electrochemical Impedance Spectroscopy

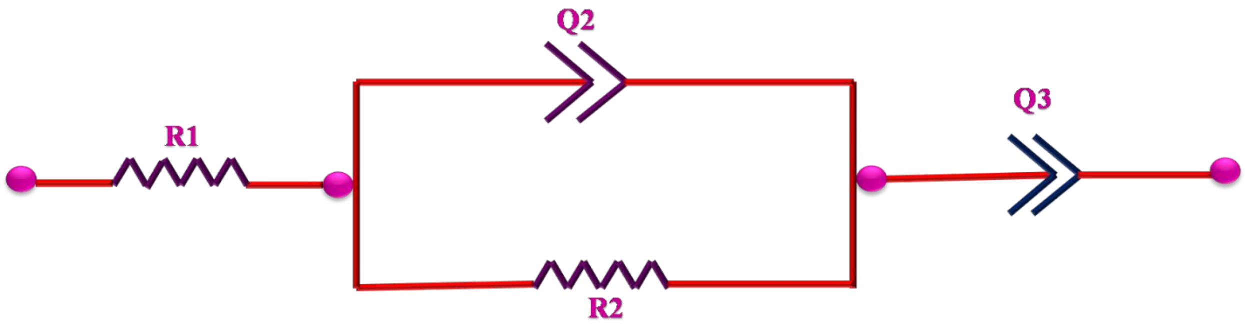

Figure S1. Equivalent circuit diagram of C_3N_4- B, C_3N_4- H_2SO_4, C_3N_4- HNO_3, and C_3N_4- HCl system.

	R_1 (Ohm)	Q_2 (mF)	a_2	R_2 (Ohm)	Q_3 (mF)	a_3
C_3N_4 – B – Before cycling	1.36	1.88	0.8439	15.49	14.56	0.6558
C_3N_4 – B – After cycling	1.37	3.42	0.7362	85.86	1.80	0.7815
C_3N_4 – H_2SO_4 – Before cycling	1.41	1.97	0.7255	31.18	109.8	0.7264
C_3N_4 – H_2SO_4 – After cycling	2.08	2.03	0.7644	67.42	1.38	0.8416
C_3N_4 – HNO_3 – Before cycling	0.36	3.95	0.7104	89.8	17.22	0.5492
C_3N_4 – HNO_3 – After cycling	3.09	1.10	1.000	32.32	1.18	0.8395
C_3N_4 – HCl – Before cycling	0.78	11.47	0.8516	2.71	0.30	0.804
C_3N_4 – HCl – After cycling	1.48	22.25	0.7183	169.1	2.18	0.8646

Table S3. Fitted equivalent circuit elements for C_3N_4- B, C_3N_4- H_2SO_4, C_3N_4- HNO_3, and C_3N_4- HCl before & after cycling test.

S2. High mass charge transfer:

The high mass charge transfer is usually associated with the measurement is performed in the presence of a redox couple (Ox/Red) by introducing small sinusoidal voltage perturbations superimposed on a direct current (DC) potential that aligns with the standard potential (E°) of the redox reaction. The total current flowing through R1 can be separated into two components: the first component pertains to the charging and discharging of the electric double layer capacitance, while the second component is associated with the faradaic process. This faradaic process encompasses both the kinetics of the redox reaction (charge transfer resistance) and the diffusion of the redox species (Warburg impedance) toward the surface of the working electrode.

In this context, the electrochemical system undergoes a reversible redox reaction with an electrolyte. This process is typically associated with changes in the charge transfer resistance (R2), which is a result of the kinetics of the redox reaction in conjunction with the capacitance Q. The value of R2 is contingent on the number of electrons liberated during the redox reaction and is frequently linked to the mass transfer involved in the electrochemical process. In our specific scenario, the acid-treated material exhibits a greater release of electrons during the charge-discharge cycle compared to the pristine material. This directly affects the values of R and Q within the system. The term "high mass charge" is employed to describe the charge storage characteristics associated with the redox reaction[1].

S3. Electrochemical Analysis

The cyclic voltammogramprofile of pristine C_3N_4-B, as well as various acid-treated C_3N_4-H_2SO_4, C_3N_4-HNO_3, and C_3N_4-HCl at different scan rates ranging from 5 to 150 mV/s in the potential range of 0 to 0.4V is depicted in Figures S2a, S3a, and S4a & S5a. Figure S2b, S3b, S4b &S5b show the charge-discharge profile of C_3N_4- B, C_3N_4- H_2SO_4, C_3N_4- HNO_3, and C_3N_4-HCl at different current densities. Figures S2d, S3d, S4d & S5d dillustrate the plot of specific capacitance against various current densities of C_3N_4- B, C_3N_4- H_2SO_4, C_3N_4- HNO_3, and C_3N_4- HCl. The Nyquist plot for C_3N_4- B, C_3N_4- H_2SO_4, C_3N_4- HNO_3, and C_3N_4- HCl before and after cycling test is presented in Figures S2c, S3c, S4c &S5c.

From the cyclic voltammogram profile, pristine C_3N_4-B exhibits redox peaks due to its surface functional groups and pseudo-capacitive behaviour. The increase in current densities with respect to the scan rate indicates that the reaction mechanism is diffusion-controlled. The specific capacitance was calculated using equation (1) with the GCPL technique and the maximum specific capacitance of 107 F/g was achieved at a current density of 3 A/g. The interfacial reaction between the electrode and electrolyte was studied using the Nyquist plot, which was fitted with resistive and capacitive elements. The charge transfer resistance increased after cycling, reflecting the formation of charged ions and low intrinsic resistance due to the material's good conductivity.

The cyclic voltammogram profile of sulfuric acid-treated C_3N_4 shows high current density with significant redox peaks, indicating the exfoliation of carbon nitride sheets into layers. This leads to notable charge storage contribution and pseudo-capacitive behavior. The maximum specific capacitance of 175 F/g was achieved at a current density of 5 A/g, as calculated from the charge-discharge studies. The Nyquist plot was fitted with equivalent circuit elements consisting of a resistor and capacitor to study the interfacial reaction mechanism. The

value of Q_3 is quite high before cycling, demonstrating the contribution of the pseudo-capacitive element to charge storage. However, it decreases after cycling due to the reduction of electrochemical species and increased intrinsic resistance.

The CV profile of nitric acid-treated C_3N_4 shows that the separated few layers of stacked carbon nitride exhibit pseudo-capacitive behavior and undergo redox reactions with the electrolyte. The specific capacitance was calculated using charge-discharge studies. The maximum specific capacitance of 75 F/g was achieved at a current density of 5 A/g. The Nyquist plot was used to study the interfacial reaction, fitted with equivalent circuit elements consisting of resistive and capacitive components. The material has a low internal resistance, indicating good conductivity and the exfoliation of layers into few layers. The charge transfer resistance decreases after cycling due to the reduction of active species and breakage of interlayer.

The cyclic voltammogram profile of hydrochloric acid-treated C_3N_4 shows that monoprotic hydrochloric acid (H^+Cl^-) dissociates the stacked interlayer into a few nano layers. This leads to high current density and redox peaks at low scan rates, and enhances the charge storage mechanism. The maximum specific capacitance of 761 F/g was achieved at a current density of 20 A/g. The Nyquist plot is used to study the interfacial reaction with a fitted equivalent circuit diagram consisting of resistive and capacitive elements listed in the table. The intrinsic resistance value is very low even after cycling, which clearly indicates that the exfoliation of stacked carbon nitride into few layers leads to better ionic conductivity and high specific capacitance with excellent cyclic stability, even after 10,000 cycles. The value of Q2 is quite high both before and after cycling, which signifies an effective mass charge transfer due to increased wettability.

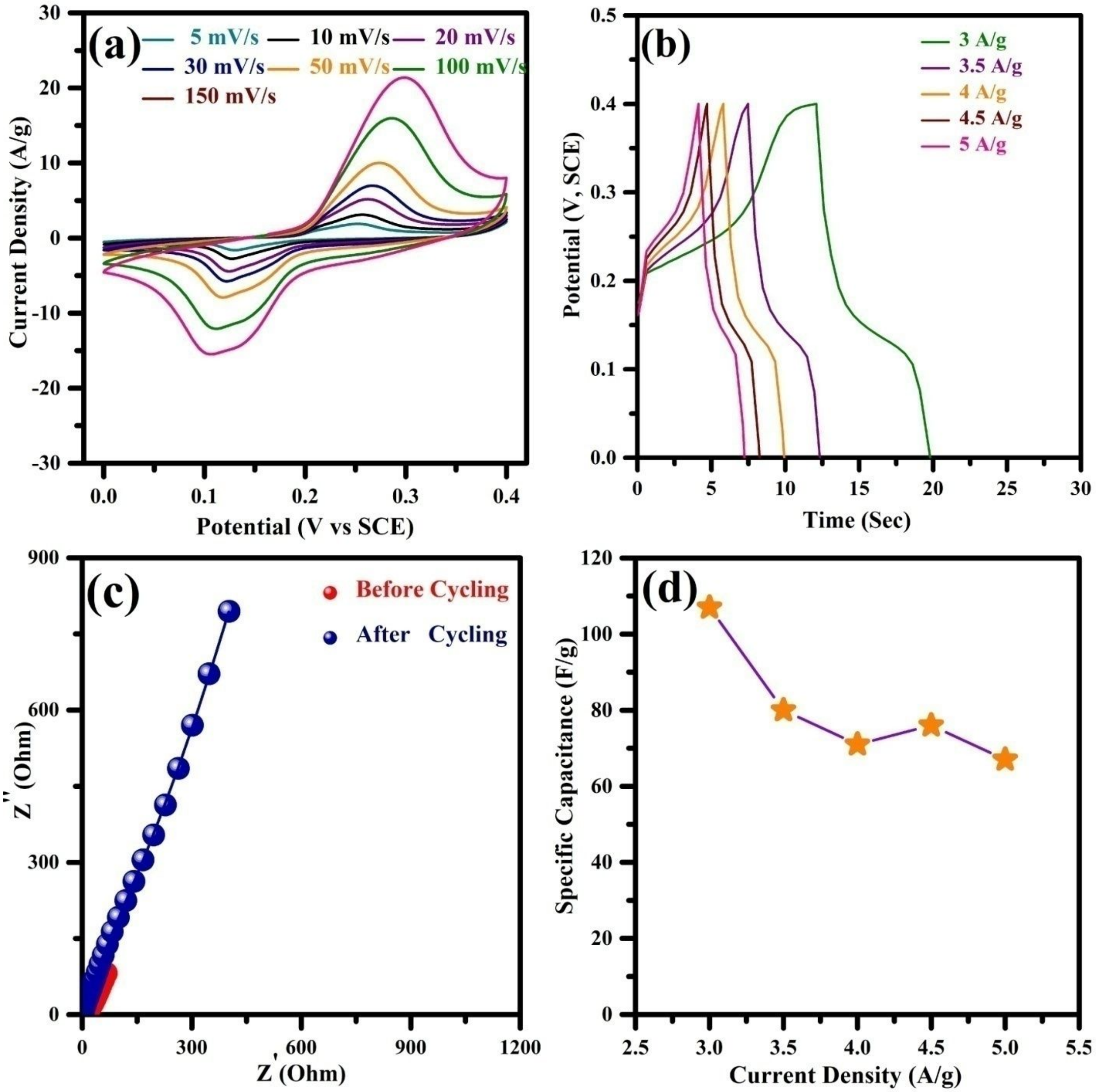

Figure S2. (a) Cyclic Voltammogram profile of Pristine C_3N_4- B system at various scan rates. (b) CD profile of PristineC_3N_4- B system at various current densities. (c) Nyquist plot of Pristine C_3N_4 (C_3N_4- B) system before and after cycling test. (d) A plot of specific capacity vs. current density at various current densities.

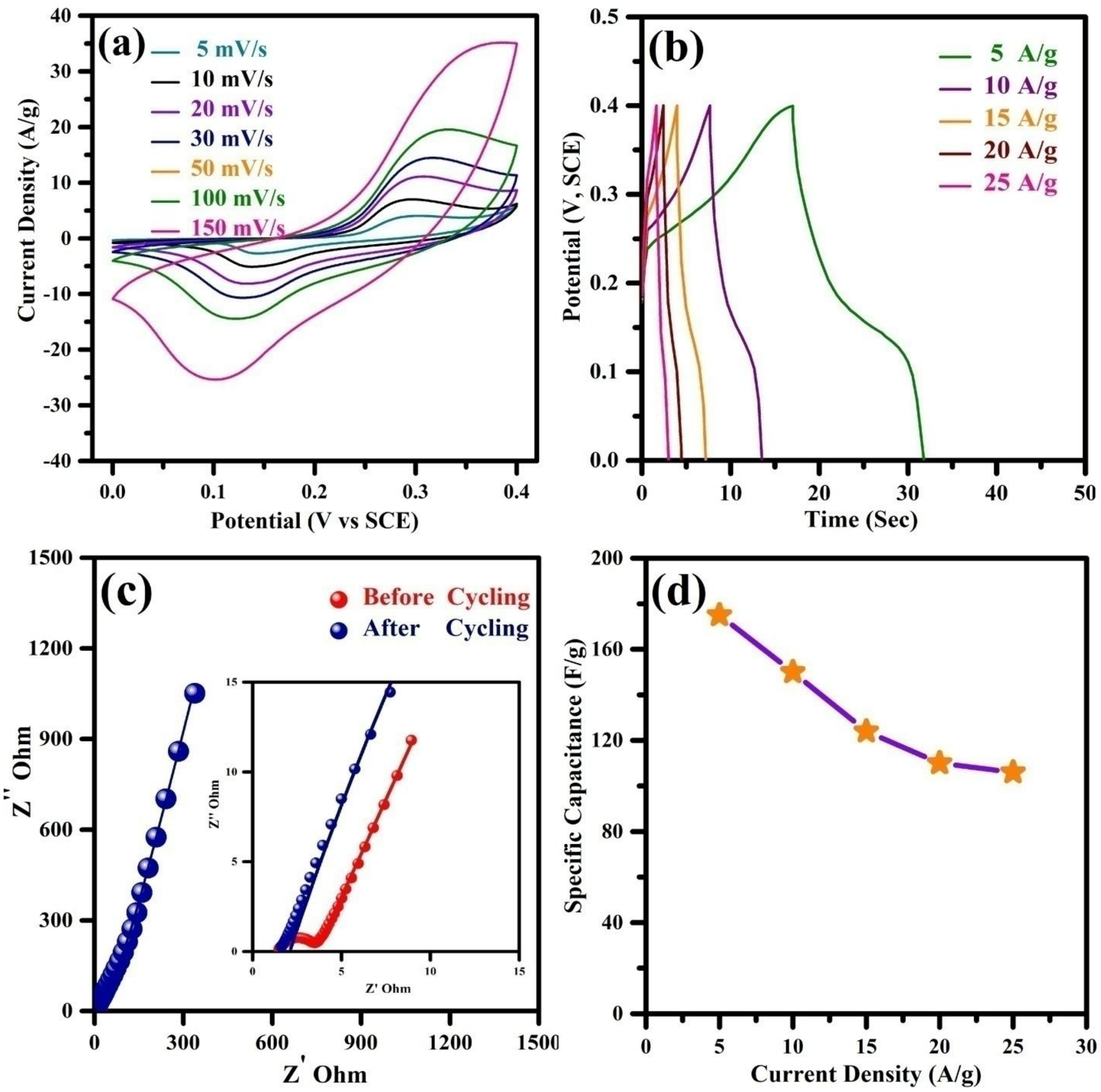

Figure S3. (a) Cyclic Voltammogram profile of C_3N_4- H_2SO_4 system at various scan rates. (b) CD profile of C_3N_4- H_2SO_4 system at various current densities. (c) Nyquist plot of C_3N_4- H_2SO_4 system before and after cycling test. (d) A plot of specific capacity vs. current density at various current densities.

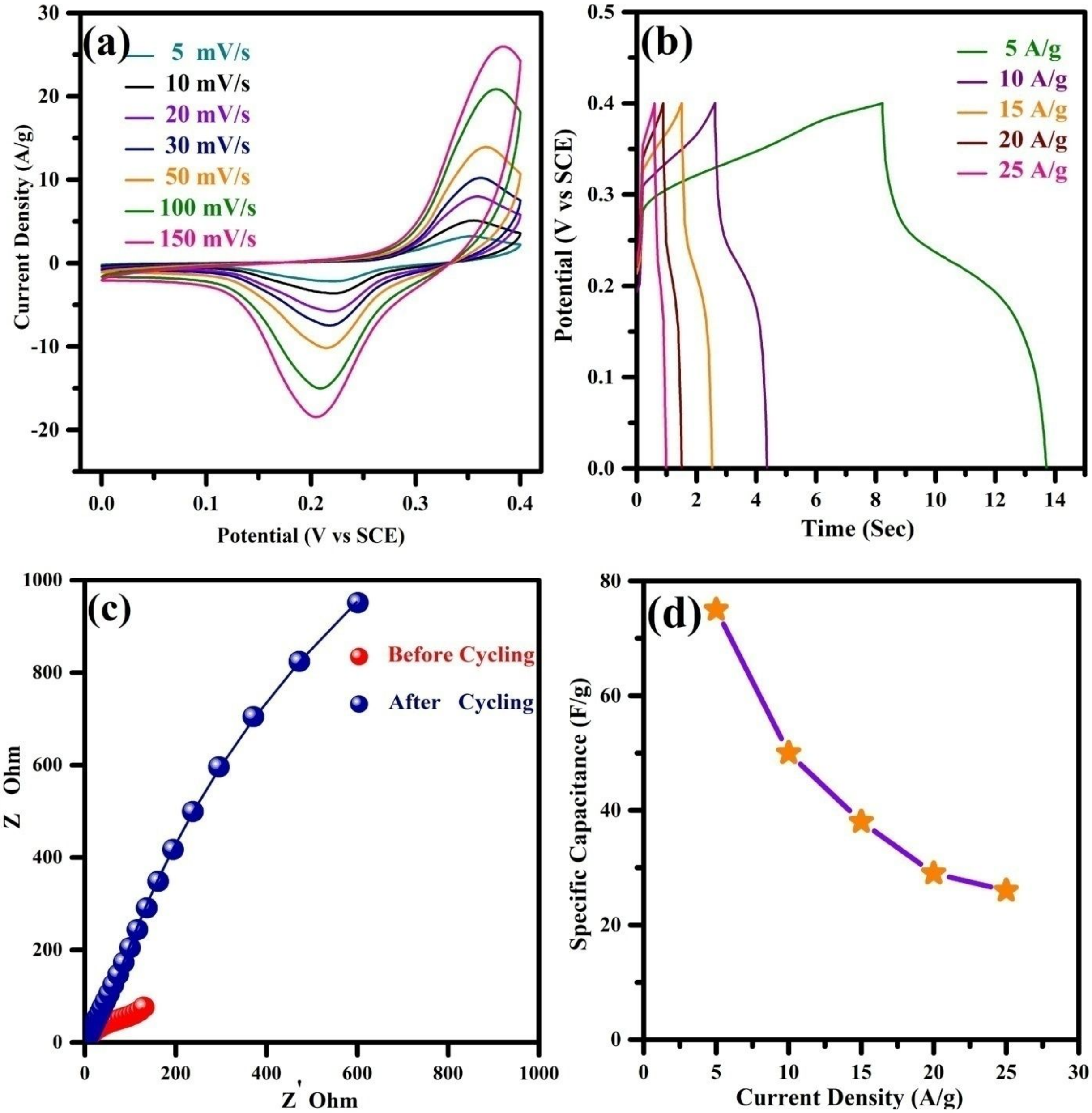

Figure S4. (a) Cyclic Voltammogram profile of C_3N_4- HNO_3 system at various scan rates. (b) CD profile of C_3N_4– HNO_3 system at various current densities. (c) Nyquist plot of C_3N_4- HNO_3 system before and after cycling test. (d) A plot of specific capacity vs. current density at various current densities.

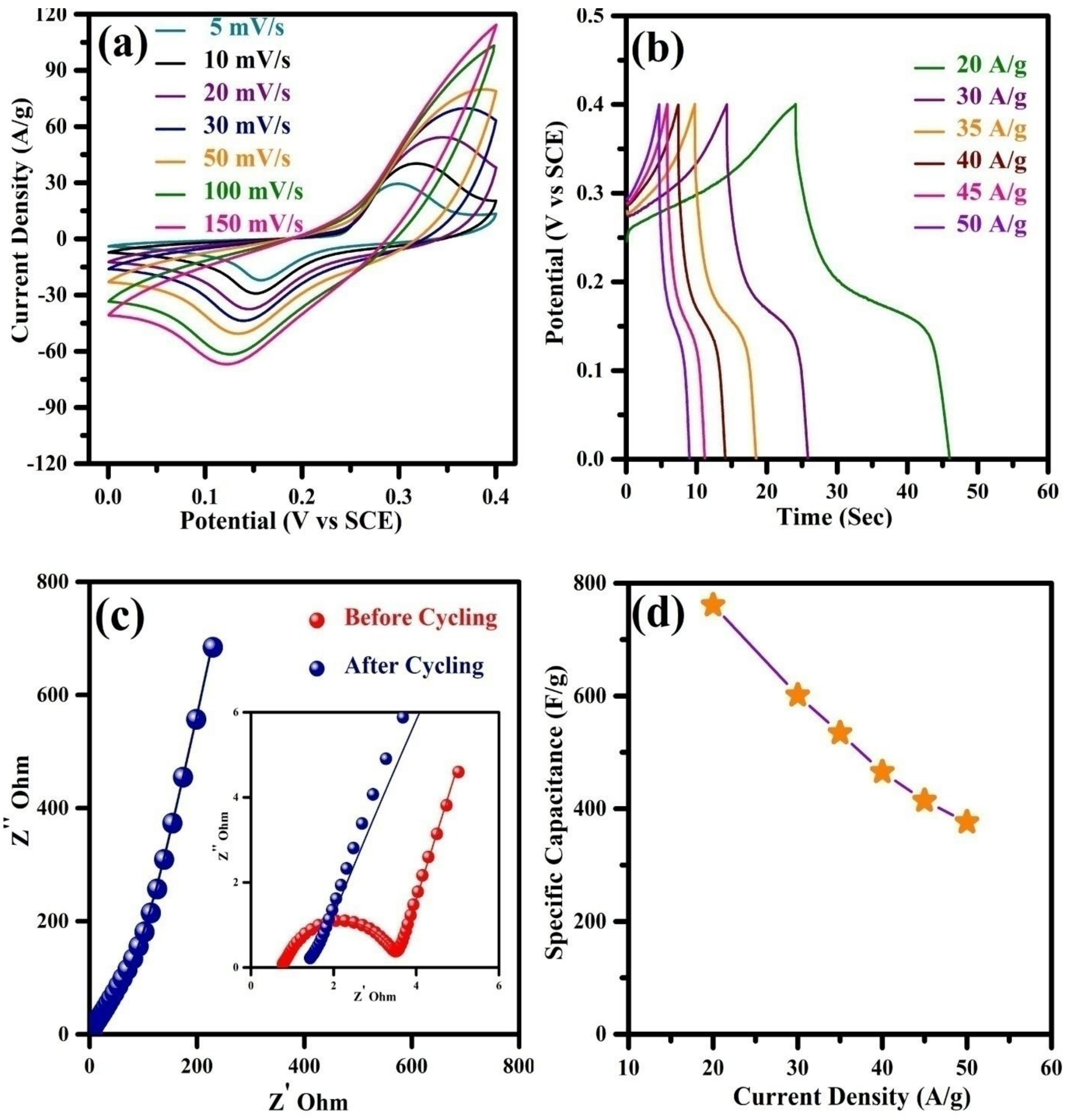

Figure S5. (a) Cyclic Voltammogram profile of C_3N_4- HCl system at various scans rates. (b) CD profile of C_3N_4- HCl system at various current densities. (c) Nyquist plot of C_3N_4- HCl system before and after cycling test. (d) A plot of specific capacity vs. current density at various current densities.

S4. Absorption behavior:

Figure S6 represents the UV-Vis absorption spectroscopy for pristine and various acid-treated C_3N_4. It is evident that protonation has a strong influence on the optical absorption property. The red shift in the absorption edge implies extended wavelength absorption of the material. Among the pristine and other treated acids, HCl shows an extended absorption band edge with high absorbing intensities due to the effective etching of the layers. It also accentuates that hydrochloric acid exfoliated the interlayer more efficiently. Nitric acid shows a slight change due to the weak etching effect of the acid compared to sulfuric acid and hydrochloric acid.

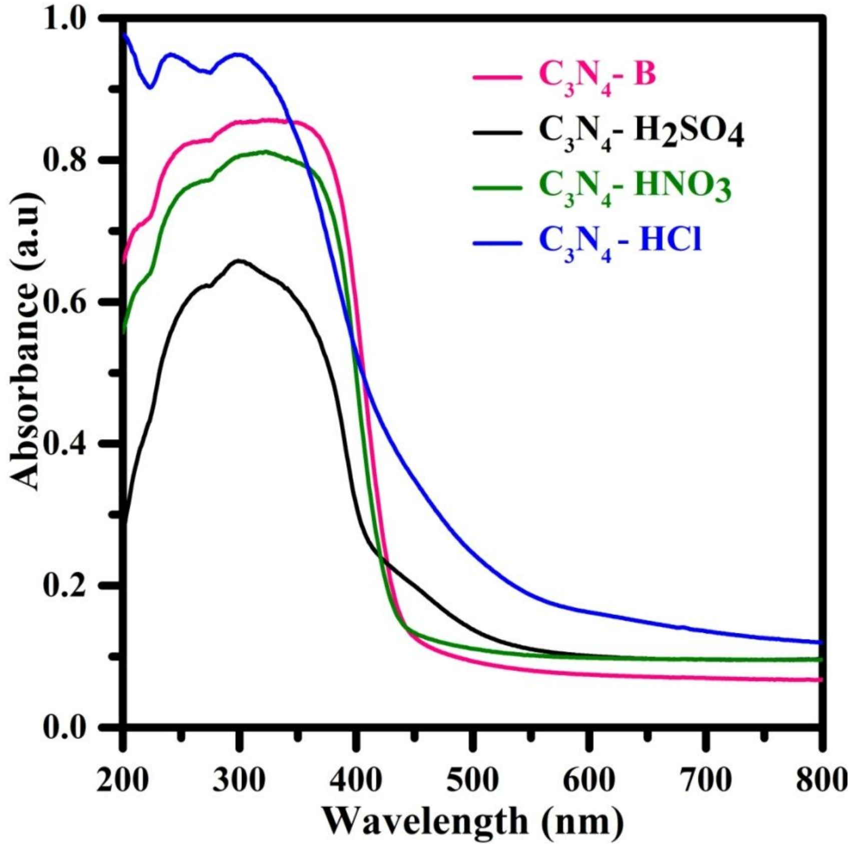

Figure S6. UV – DRS spectrum of pristine C_3N_4- B and various acid treated C_3N_4- H_2SO_4, C_3N_4- HNO_3, and C_3N_4- HCl.

S5. Surface Morphology

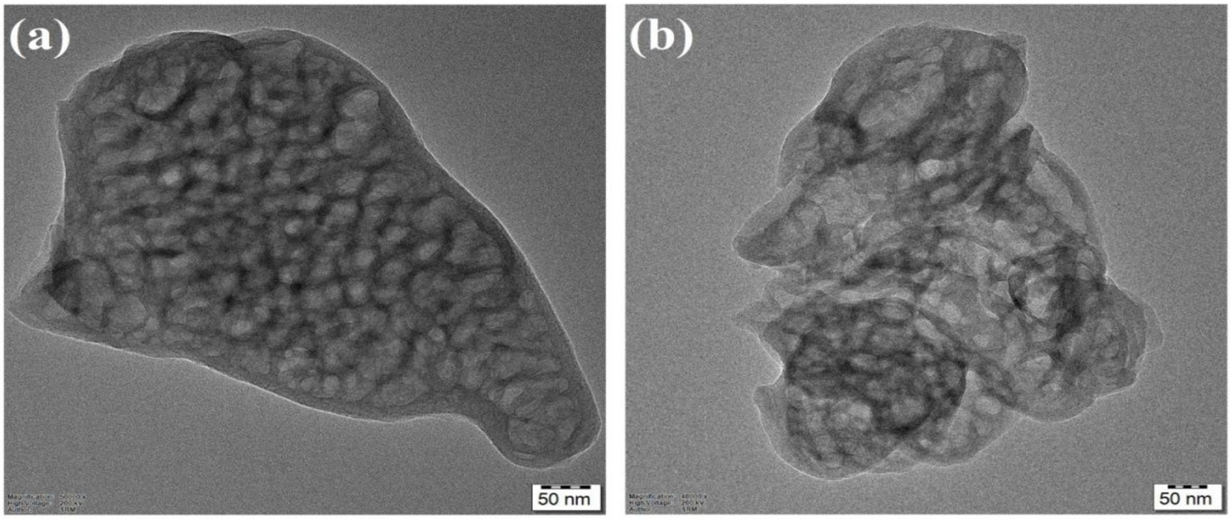

Figure S7. HR TEM image (a) C_3N_4- B at 50 nm scale. (b) C_3N_4- HCl at 50 nm scale.

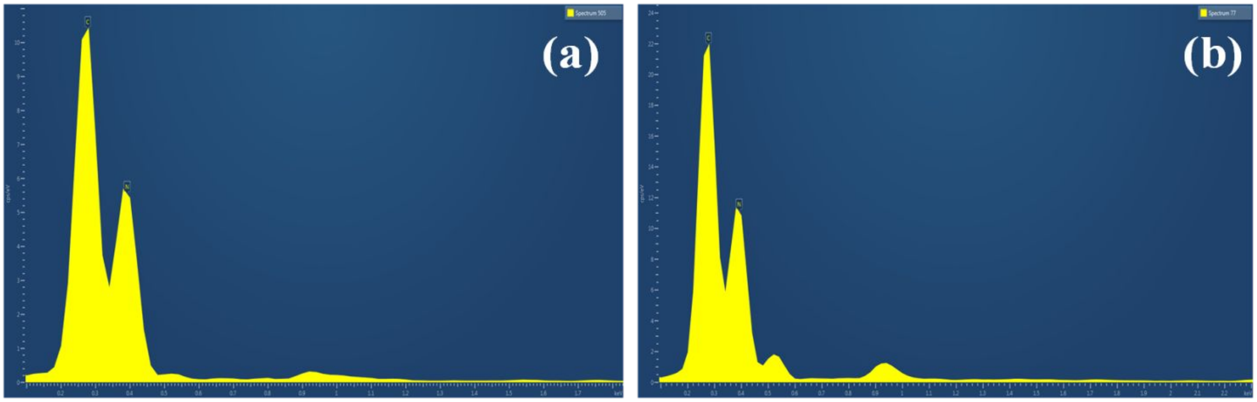

Figure S8. Elemental composition of (a) C_3N_4- B. (b)C_3N_4- HCl obtained through TEM analysis.

	Element	Wt %	Atomic %
C_3N_4- B	C	61.67	65.24
	N	38.33	34.76
C_3N_4- HCl	C	60.10	63.73
	N	39.90	36.27

Table S4. Atomic & Weight Percentage of C_3N_4- B, C_3N_4- HCl obtained through TEM EDAX analysis.

S6. Elemental survey of O1s:

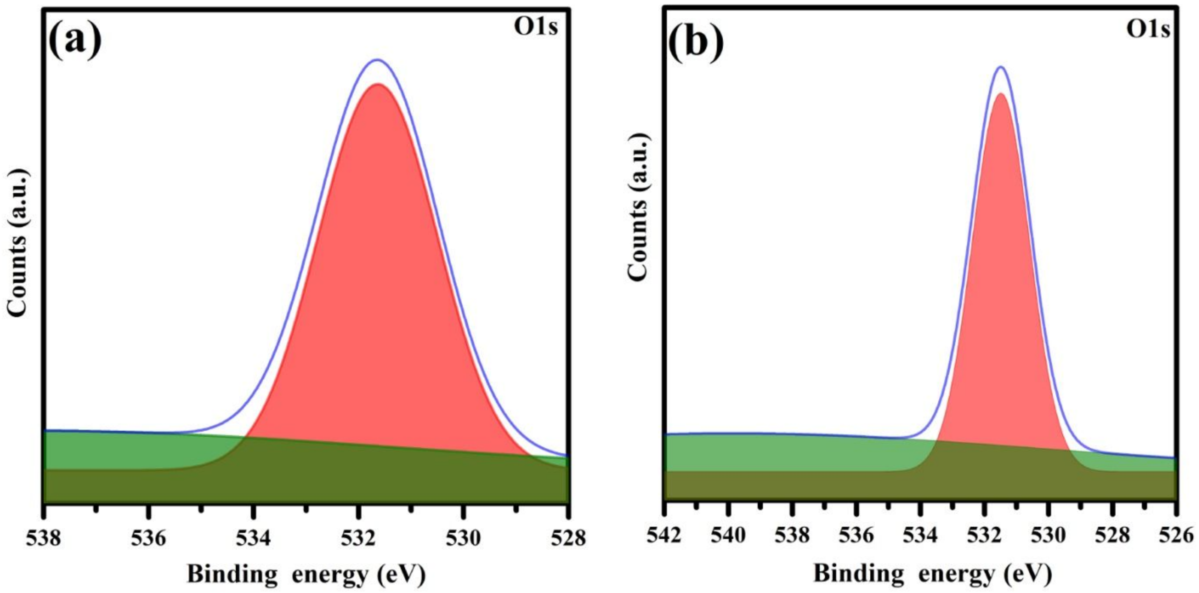

Figure S9. O1s region of (a) C_3N_4 - B and (b) C_3N_4- HCl.

	Element	Atomic %
C_3N_4- B	C	35.30
	N	64.70
C_3N_4- HCl	C	34.27
	N	65.73

Table S5. Atomic of C_3N_4- B, C_3N_4- HCl obtained through XPS elemental analysis.

	Parameters	1 A/g	2 A/g	3 A/g	10 A/g	15 A/g	
C_3N_4- HCl (1.2 V, voltage window)	Specific Capacitance (F/g)	175	40	15	8.33	6.25	
	Energy Density($WhKg^{-1}$)	35	9	3	1.66	1.2	
	Power Density(WKg^{-1})	600	1.44 K	1.80 K	5.76 K	9 K	
	Parameters	3 A/g	5 A/g	10 A/g	15 A/g	20 A/g	25 A/g
C_3N_4- HCl (1.8 V, voltage window)	Specific Capacitance (F/g)	3	2.73	2.055	1.641	0.77	0.277
	Energy Density($WhKg^{-1}$)	1.35	1.22	328.5 m	88 m	31 m	1.2 m
	Power Density(WKg^{-1})	4.96 K	4.93 K	1.621 K	1.60 K	1.59 K	1.58 K

Table S6. The calculated Specific Capacitance, Energy Density and Power Density of the fabricated symmetric device

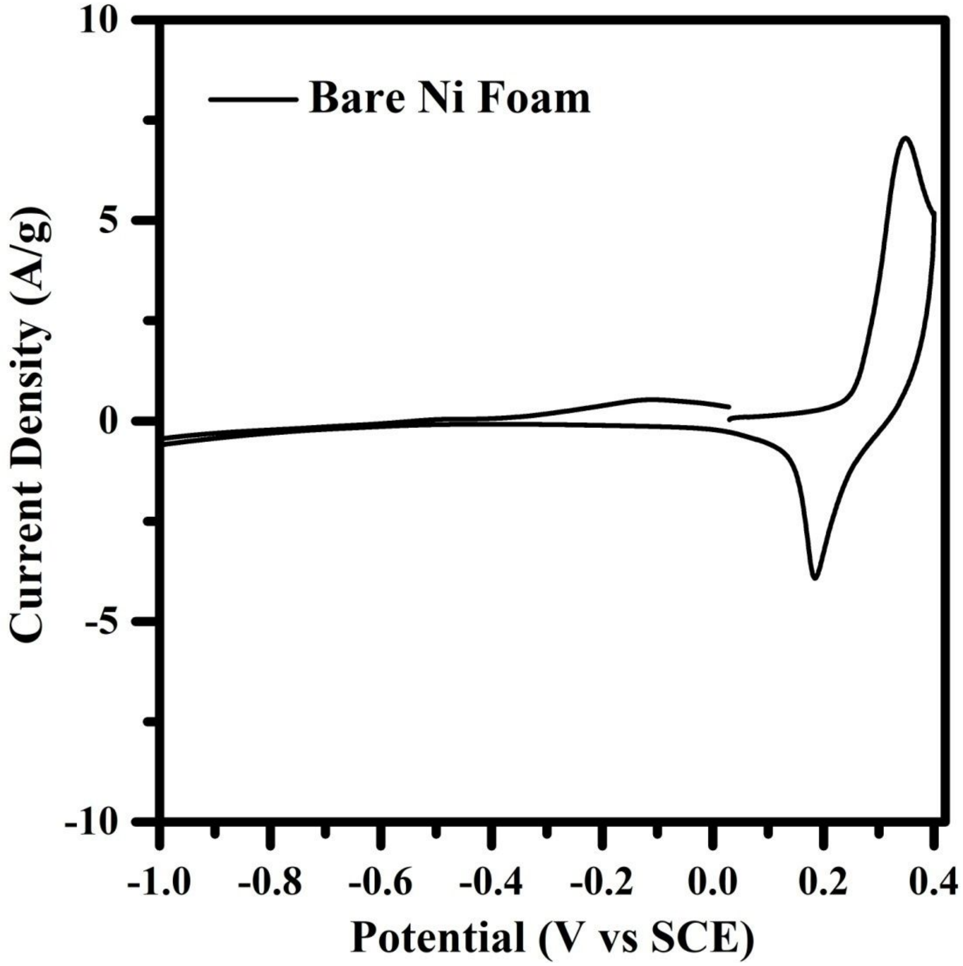

Figure S10. Cyclic Voltammogram profile of bare Ni foam in 6M KOH solution.

S7. Structural behaviour:

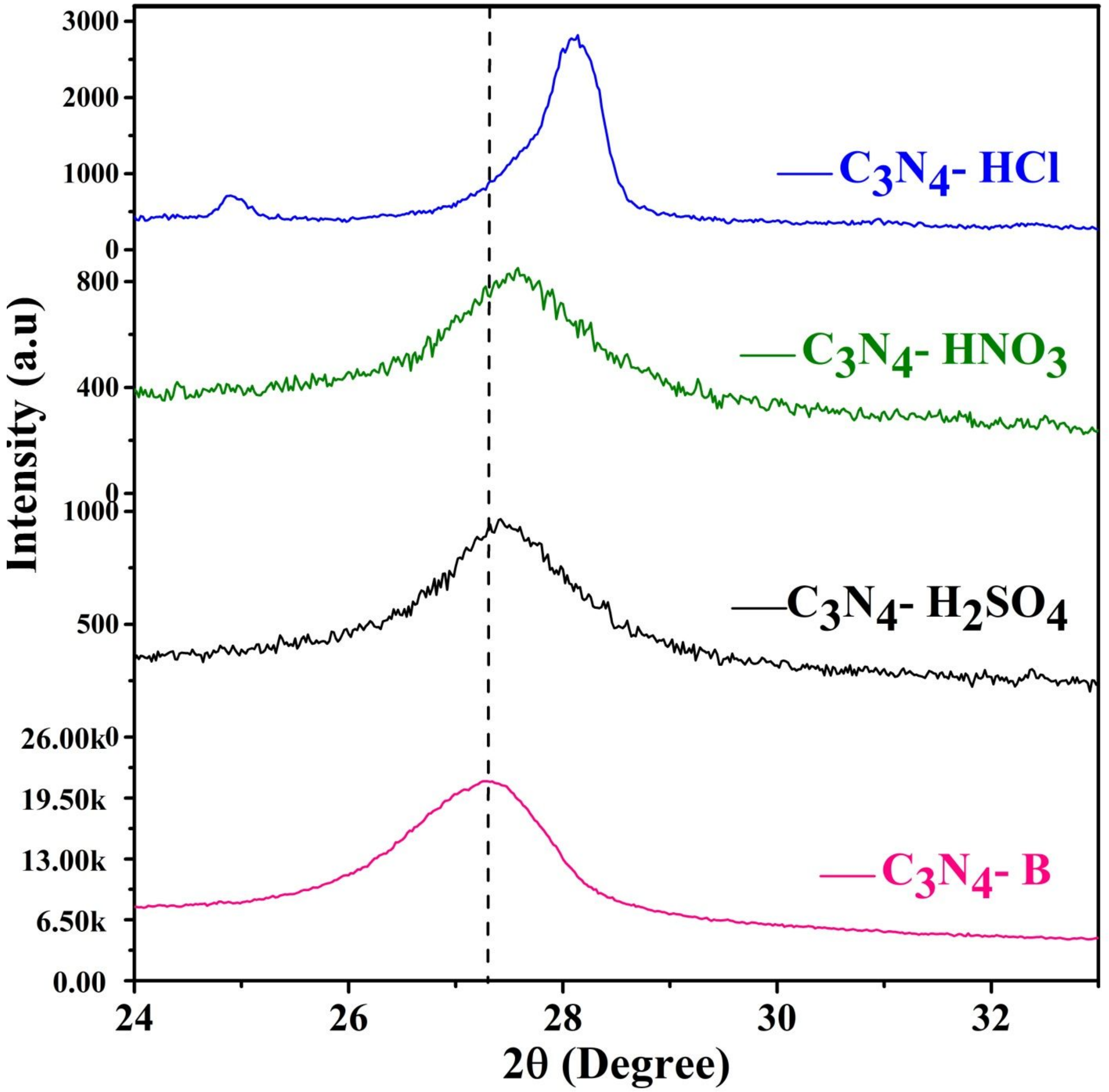

Figure S11. X ray diffraction pattern of C_3N_4- B, C_3N_4- H_2SO_4, C_3N_4- HNO_3, and C_3N_4- HCl.

References

(1) Lazanas, A. C.; Prodromidis, M. I. Electrochemical Impedance Spectroscopy─A Tutorial. *ACS Measurement Science Au* **2023**, *3* (3), 162-193. DOI: 10.1021/acsmeasuresciau.2c00070.
